# Metformin and histone deacetylase inhibitor based anti-inflammatory nanoplatform for epithelial-mesenchymal transition suppression and metastatic tumor treatment

**DOI:** 10.1186/s12951-022-01592-6

**Published:** 2022-08-31

**Authors:** Tianze Jiang, Laozhi Xie, Songlei Zhou, Yipu Liu, Yukun Huang, Ni Mei, Fenfen Ma, Jingru Gong, Xiaoling Gao, Jun Chen

**Affiliations:** 1 Shanghai Pudong Hospital & Department of Pharmaceutics, School of Pharmacy, Fudan University, Lane 826, Zhangheng Road, Shanghai, 201203 People’s Republic of China; 2 Key Laboratory of Marine Drugs, Ministry of Education, Shandong Key Laboratory of Glycoscience and Glycotechnology, School of Medicine and Pharmacy, Ocean University of China, 5 Yushan Road, Qingdao, 266003 People’s Republic of China; 3 Department of Pharmacology and Chemical Biology, Shanghai Jiao Tong University School of Medicine, 280 South Chongqing Road, Shanghai, 200025 People’s Republic of China; 4 Shanghai Center for Drug Evaluation and Inspection, Lane 58, HaiQv Road, Shanghai, 201210 People’s Republic of China; 5 Department of Pharmacy, Shanghai Pudong Hospital, Fudan University Pudong Medical Center, 2800 Gongwei Road, Shanghai, 201399 People’s Republic of China; 6 Key Laboratory of Smart Drug Delivery, Ministry of Education, School of Pharmacy, Fudan University, Lane 826, Zhangheng Road, Shanghai, 201203 People’s Republic of China

**Keywords:** Tumor metastasis, Anti-inflammation, Epithelial-mesenchymal transition, Metformin, Histone deacetylase inhibitor, Nanoplatform

## Abstract

Epithelial-mesenchymal transition (EMT), a differentiation process with aberrant changes of tumor cells, is identified as an initial and vital procedure for metastatic processes. Inflammation is a significant inducer of EMT and provides an indispensable target for blocking EMT, however, an anti-inflammatory therapeutic with highlighted safety and efficacy is deficient. Metformin is a promising anti-inflammatory agent with low side effects, but tumor monotherapy with an anti-inflammation drug could generate therapy resistance, cell adaptation or even promote tumor development. Combination therapies with various anti-inflammatory mechanisms can be favorable options improving therapeutic effects of metformin, here we develop a tumor targeting hybrid micelle based on metformin and a histone deacetylase inhibitor propofol-docosahexaenoic acid for efficient therapeutic efficacies of anti-inflammatory drugs. Triptolide is further encapsulated in hybrid micelles for orthotopic tumor therapies. The final multifunctional nanoplatforms (HAOPTs) with hyaluronic acid (HA) modification can target tumor efficiently, inhibit tumor cell EMT processes, repress metastasis establishment and suppress metastatic tumor development in a synergistic manner. Collectively, the results afford proof of concept that the tumor targeting anti-inflammatory nanoplatform can provide a potent, safe and clinical translational approach for EMT inhibition and metastatic tumor therapy.

**Supplementary Information:**

The online version contains supplementary material available at 10.1186/s12951-022-01592-6.

## Background

Metastasis is a critical reason of high cancer mortality in clinic [1, 2]. Conventional therapeutics such as chemotherapy and surgery are exploited for efficient orthotopic tumor therapies but exhibit low effectiveness against metastasis or even facilitate metastasis formation [3–5]. During metastasis processes, epithelial-mesenchymal transition (EMT), a differentiation process of epithelial cells to mesenchymal-like cells, has been recognized as an initial and necessary procedure for tumor cell invasion in primary tumor tissues [6]. Epithelial-like tumor cells can lose tight intercellular junctions, remodel their cytoskeleton, alter gene expression and finally obtain an invasive phenotype, promoting the migration and intravasation of tumor cells. Characteristic changes within EMT processes include inhibition of E-cadherin expression as well as up-regulation of metalloproteinase-9 (MMP-9) and Collagen I, etc.[7]. In addition, the metastasis formation resembles the tissue repair process superficially. Invasive tumor cells derived from EMT request self-renewal and differentiation abilities similar to stem cells involved in tissue repair processes, generating cancer stem cell (CSC)-like phenotype and ensuring cell survival for tumor cell dissemination and metastasis formation [7–9]. Consequently, the EMT process in primary tumors could be considered as a crucial target for metastasis prevention.

EMT processes are regulated by a complicated network with diverse pathways that interplay with each other. Currently, anti-EMT drugs that block certain molecular interactions, such as the TGF-β-receptor I kinase inhibitor Galunisertib, are less effective, probably due to the large number of crosstalk and substitution mechanisms that can induce the EMT process [10, 11]. New strategies focusing on upstream mechanisms of EMT would be essential for effective suppression of EMT. Inflammation is a hallmark that induces EMT in primary tumors [12, 13]. Tumor inflamed microenvironments can trigger multi-pathways, such as nuclear factor-κB (NF-κB) pathway or M2 macrophage polarization, to induce abnormal changes in EMT process as mentioned above [12–18]. Therefore, the inflammatory microenvironment could be a potential target for the inhibition of EMT. However, traditional anti-inflammation drugs such as dexamethasone are always accompanied by side effects such as strong immunosuppression [19, 20]. New anti-inflammatory agents with low side effects were expected to provide a promising method to efficiently prevent metastasis by interfering multi-events in EMT.

Among the drugs with anti-inflammation activity, metformin, a widely applied drug of type II diabetes, presents low toxicity and side effects with doses ranging from 500 to 2500 mg a day [21]. During the tumor treatments, metformin can be considered as a valid strategy in inflammation repression to reduce the possibility of EMT incident via AMP-activated protein kinase (AMPK) activation [22–24]. However, tumor monotherapy with an anti-inflammation drug could prompt cell adaptation, treatment resistance or even tumor development [25]. Thus, rational targeted combination treatments with diverse anti-inflammatory mechanisms could realize the therapeutic potential of anti-inflammatory metformin for metastasis prevention. Histone deacetylases (HDACs), a class of enzymes overexpressed in numerous tumors [26], can alter the transcription of genes by degrading acetyl groups of transcription factors and chromatin histones [27, 28]. Various transcription factors participate in several inflammatory pathways, for instance, NF-κB and signal transducers and activators of transcription (STATs). Since the activity of inflammatory transcription factors is regulated by deacetylation of HDACs, the inhibition of HDAC may be characterized for their anti-inflammation potency [29, 30]. Accordingly, we expected that the application of metformin combined with a HDAC inhibitor would efficiently suppress the EMT process and tumor metastasis by modulating inflammatory tumor microenvironments.

**Fig. 1:**
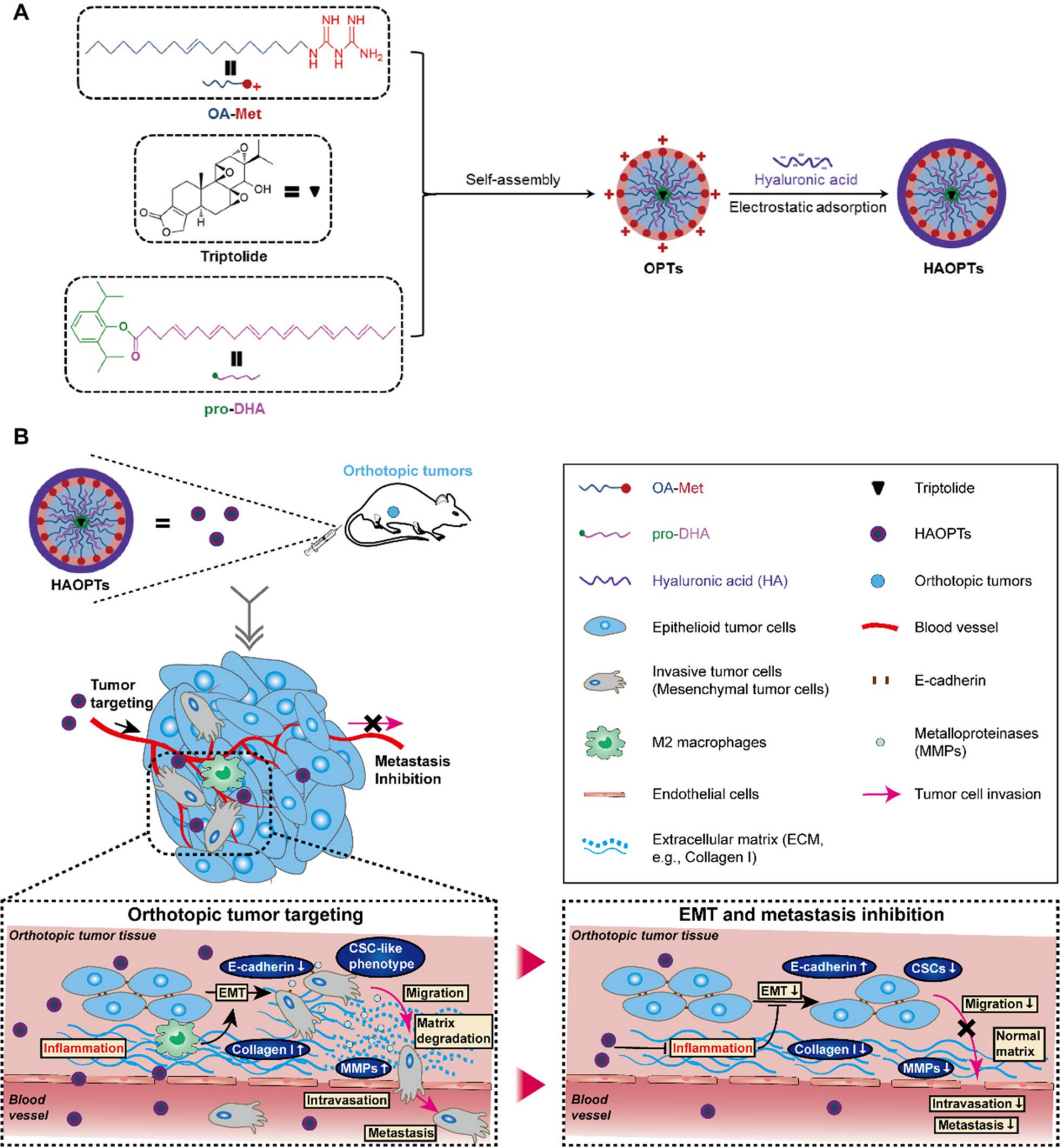
Schematic illustration of hyaluronic acid-cloaked micelles (HAOPTs) for the treatment of metastatic tumors. **A** Schematic protocol of the HAOPTs preparation. **B** HAOPTs target orthotopic tumor tissues and suppress characteristic abnormal alterations in the EMT process, inhibiting further metastasis establishment

In order to prove the assumption that targeted multi-drugs with different anti-inflammatory mechanisms would afford an efficient approach for inhibition of EMT, here we developed an anti-inflammation based tumor targeting nanoplatform for the treatment of EMT and tumor metastasis (Fig. 1) [31, 32]. Hybrid micelles were prepared by a novel amphiphilic derivative of metformin with oleate carbon chain (OA-Met) and a HDAC inhibitor pro-DHA (propofol-docosahexaenoic acid) for efficient therapeutic effects of anti-inflammatory agents. In addition, an effective anti-metastasis nanoplatform should possess the inhibitory ability of orthotopic tumors, we expected to encapsulate the natural extract triptolide in hybrid micelles for multifunctional treatments of metastatic tumors [33–35]. The obtained triptolide loaded hybrid micelles (OPTs) were finally surface modified with hyaluronic acid (HA) that facilitate the tumor targeting effect (Fig. 1A) [36, 37]. The resulted micelles (HAOPTs) could target tumor tissues effectively, suppress tumor cell EMT processes, repress primary tumor development and inhibit metastasis formation in a synergistic manner. Collectively, our study firstly provided proof of concept that the inhibition of EMT processes by tumor targeting anti-inflammatory nanoplatform would offer a potent, safe and clinical translational strategy for metastasis therapy.

## Materials and methods

### Materials

Propofol, dicyclohexylcarbodiimide (DCC), 4-dimethylaminopyridine (DMAP), butylated hydroxytoluene (BHT), anhydrous dichloromethane, dextran sulfate and phosphotungstic acid were purchased from Aladdin Co., Ltd (Shanghai, China). Docosahexaenoic acid (DHA), coumarin-6, 1,1’-dioctadecyl-3,3,3’,3’-tetramethyl indotricarbocyanine iodide (DiR) and 3-(4,5-dimethylthiazol-2-yl)-2,5-diphenyltetrazolium bromide (MTT) were obtained from Sigma-Aldrich (St. Louis, MO, USA). Pyrene was provided by J&K scientific Co., Ltd (Shanghai, China). Triptolide was purchased from Biopurify Phytochemicals Ltd (Chengdu, China). HA (MW = 30,000) was provided by Bloomage Biotechnology Co., Ltd (Jinan, China). D-luciferin potassium was bought from PerkinElmer Inc. (Waltham, MA, USA). Interleukin-13 (IL-13) was purchased from Sinobio Co., Ltd (Shanghai, China). Matrigel was acquired from BD Bioscience (San Jose, CA, USA). Crystal violet staining solution was obtained from Beyotime Biotechnology Co., Ltd (Shanghai, China). Phycoerythrin (PE) labeled anti-CD24 antibody, PE labeled anti-CD206 antibody and fluorescein isothiocyanate (FITC) labeled anti-CD44 antibody were provided by eBioscience. Anti-Histone H3 (acetyl K9) antibody, anti-E-cadherin antibody, anti-MMP-9 antibody and anti-Collagen I antibody were bought from Abcam (Cambridge, MA, USA). Anti-phospho-AMPK (p-AMPK, Thr172) antibody was purchased from Cell Signaling Technology (Danvers, MA, USA). In situ cell death detection kit was obtained from Roche (Basel, Switzerland). All the other chemical reagents and solvents were acquired from Sinopharm Chemical Reagent Co., Ltd (Shanghai, China).

Dulbecco's modified eagle medium (DMEM), RPMI 1640 medium, certified fetal bovine serum (FBS), penicillin–streptomycin stock solutions and trypsin–EDTA (0.25%) were obtained from Invitrogen (Carlsbad, CA, USA). DMEM/F12 medium, B-27 supplement (50 ×), basic fibroblast growth factor (bFGF) and epidermal growth factor (EGF) were provided by Gibco (USA).

### Cell lines and animals

Murine RAW264.7 macrophages cells, 4T1 cells and 4T1-Luc cells were provided by Chinese Academy of Sciences Cell Bank (Shanghai, China). RAW264.7 cells were cultured in DMEM medium, 4T1 cells and 4T1-Luc cells were cultured in RPMI 1640 medium, supplemented with 10% FBS (v/v), 100 mg/mL streptomycin and 100 U/mL penicillin at 37 °C in a humidified atmosphere with 5% CO_2_.

Female Balb/c mice (20 ± 2 g) and female Spraguee-Dawley (S-D) rats (200 ± 10 g) were purchased from BK Lab Animal Ltd. (Shanghai, China) and kept under standard housing condition (25 ± 1 °C with access to food and water ad libitum).

### Synthesis and characterization of pro-DHA

DHA (0.152 mmol) was firstly dissolved in anhydrous dichloromethane (5 mL). DCC (0.228 mmol), DMAP (0.138 mmol) and BHT (0.8 μM, an antioxidant) were added to the solution of DHA in dichloromethane, and the reaction mixture was stirred at room temperature for 2 h under nitrogen. Then a solution of propofol in anhydrous dichloromethane (0.138 mmol) was added dropwise, and the reaction mixture was stirred at reflux for 12 h under nitrogen. After the completion of reaction, the solvent was evaporated in vacuo, and the crude product was purified by column chromatography to obtain colorless oil (pro-DHA) with a yield of 53.8%. The structure of pro-DHA was verified by ^1^H-NMR (600 MHz, BRUKER) in CDCl_3_ and electron spray ionization-mass spectrometry (ESI–MS).

### Preparation and characterization of HAOPTs

OPTs were first prepared by self-assembly. Briefly, pro-DHA (2 mg) and triptolide (420 μg) were dissolved in a solution of OA-Met in ethanol (500 μL at 10 mg/mL), and the final solution was added dropwise into distilled water (1 mL) under stirring (500 rpm). The suspension was stirred for another 3 h at room temperature and ethanol was absolutely removed in vacuo at 25 °C to obtain OPTs (7.42 mg/mL). Afterward, HAOPTs were prepared by electrostatic adsorption of HA on the surface of OPTs. Briefly, aqueous OPTs suspension (216 μL) was added into HA solution (2 mL, 0.8 mg/mL) dropwise under stirring (500 rpm). The suspension was stirred for another 2 h at room temperature and HAOPTs was finally obtained.

Coumarin-6 and DiR labeled OPTs, DexOPTs or HAOPTs were prepared by the same method, whereas coumrin-6 or DiR was substituted for triptolide to dissolve in the solution of pro-DHA and OA-Met. In addition, the preparation of HAOPs (HAOPTs without triptolide) and HAOTs (HAOPTs without pro-DHA) was similar to the protocol of HAOPTs, where pro-DHA or triptolide alone was dissolved in OA-Met ethanol solution for subsequent micelle preparation.

Z-average diameter, polymer dispersity indexes (PDI) and zeta potential of micelles (OPTs, DexOPTs and HAOPTs) were detected by a dynamic light scattering detector (DLS) (Zetasizer, Nano-ZS, Malvern, UK). The stability of these micelles was determined in phosphate buffer saline (PBS) at 4 °C for 12 days and the z-average diameter was recorded at different time points.

Encapsulation efficiency (EE) and loading capacity (LC) were investigated through the high performance liquid chromatography (HPLC) method. OPTs, DexOPTs and HAOPTs were dissolved in methanol and then subjected to HPLC system. Triptolide was detected at the detector wavelength of 220 nm, and the mobile phase was a mixture of methanol and distilled water (the volume ratio of methanol to water was 45:55). Moreover, pro-DHA was identified at the detector wavelength of 214 nm, and the mobile phase was the gradient elution of distilled water and methanol, which was from 80% (methanol) to 100% (methanol) in 40 min. The EE and LC were calculated by Eqs. 1 and 2 (n = 3):

1 $${\text{EE}}\left( \% \right) = \frac{{{\text{amount}}\,{\text{of}}\,{\text{pro}} - {\text{DHA}}/{\text{triptolide}}\,{\text{in}}\,{\text{the}}\,{\text{micelles}}}}{{{\text{total}}\,{\text{amount of}}\,{\text{pro}} - {\text{DHA}}/{\text{triptolide}}\,{\text{added}}}} \times 100\%$$

2 $${\text{LC}}\left( \% \right) = \frac{{{\text{amount}}\,{\text{of}}\,{\text{pro}} - {\text{DHA}}/{\text{triptolide}}\,{\text{in}}\,{\text{the}}\,{\text{micelles}}}}{{{\text{micelles}}\,{\text{weight}}}} \times 100\%$$

The critical micelle concentration (CMC) of HAOPTs was investigated by the fluorescence probe pyrene. HAOPTs with different concentrations (1 × 10^–4^ to 2 × 10^–1^ mg/mL) were added to the tubes containing pyrene (12.5 μg), then the tubes were placed in a shaker at 37 °C for 24 h (120 rpm). After scanning pyrene by a fluorescence spectrophotometer (excitation wavelength: 335 nm, emission wavelength: 373 and 384 nm), the CMC of HAOPTs was calculated by the cross point in the plots of the fluorescence intensity ratio of 384 nm and 373 nm to the logarithm concentration of HAOPTs.

The morphology of OPTs and HAOPTs was observed by a field emission transmission electron microscope (TEM, TEM-1400 Plus Electron Microscope, Leica). Aqueous OPTs and HAOPTs suspensions (1 mg/mL) were dropped on the carbon-coated grid and water was dried under a drying light. Phosphotungstic acid solution (2%, w/v) was dropped on the dried micelles for negative staining. After 5 min, the solution was removed and the grid was subjected to a TEM for photographing.

### Cellular uptake of HAOPTs

Mouse breast tumor 4T1 cells were seeded into 96-well plates at a density of 3,000 cells per well firstly (n = 3). After incubation for 24 h, the cell culture media was replaced by free coumarin-6, coumarin-6 labeled OPTs-Cou, DexOPTs-Cou and HAOPTs-Cou (100 ng/mL for coumarin-6) and cells were incubated for 2 h in the dark. The cells were washed three times with PBS buffer, fixed with 4% paraformaldehyde (PFA) for 10 min and stained with Hoechst 33,258 at room temperature for 10 min in dark places. Finally, the cells were washed with PBS buffer for another three times, subjected to an inverted fluorescence microscope (Leica DMI 4000B, Germany) for qualitative imaging, and cellular uptake achieved quantitative analysis through a Kinetic Scan HCS Reader (version 3.1, Cellomics Inc., Pittsburgh, PA, USA).

### Pharmacokinetic study of HAOPTs

The pharmacokinetic study of HAOPTs was investigated through the following protocol. Twelve S-D rats were divided into four groups randomly (n = 3) and intravenously injected with free DiR, DiR labeled OPTs-DiR, DexOPTs-DiR and HAOPTs-DiR (270 μg/kg for DiR), respectively. Blood samples (300 μL) were collected at 0.083, 0.25, 0.5, 1, 2, 4, 6, 8, 12 and 24 h and then centrifuged at 4000 rpm for 10 min instantly. The supernatant plasma was separated from the centrifugal blood and plasma samples were stored at −20 °C.

In order to determine DiR concentration in plasma, methanol (900 μL) was added to plasma samples (100 μL) to precipitate proteins. The mixtures were vortexed for 5 min and centrifuged at 10,000 rpm for 10 min. The supernatant was separated and then subjected to microplate reader (Thermo Multiskan MK3, USA) for DiR fluorescence analysis (excitation wavelength: 748 nm, emission wavelength: 780 nm). Finally, the pharmacokinetic parameters could be calculated by Drug and Statistics (DAS) software (Version 2.0, Mathematical Pharmacology Professional Committee of China).

### Biodistribution of HAOPTs in orthotopic breast tumor mice

The distribution study of HAOPTs was performed to verify the orthotopic tumor targeting effect. 4T1 cells were inoculated into one mammary fat pad of Balb/c mice. Once the tumor grew to 100 mm^3^–200 mm^3^, the mice were divided into three groups randomly (n = 3) and injected i.v. with OPTs-DiR, DexOPTs-DiR and HAOPTs-DiR (1 mg/kg for DiR). The biodistribution of DiR labeled micelles was monitored by an in vivo IVIS spectrum imaging system (Cailper PerkinElemer, USA) at 2, 6, 12 and 24 h. After the final detection, mice were euthanized and perfused with saline and 4% PFA, followed by the harvest of major organs (heart, liver, spleen, lung and kidney) and orthotopic tumors for ex vivo imaging and semi-quantitative analysis of DiR fluorescence.

### MTT assay and combination index (CI) calculation

The tumor anti-proliferation activity of HAOPTs was assessed by MTT assay. Briefly, 4T1 cells were seeded into 96-well plates at the cell density of 1,000 cells per well (n = 3) and incubated for 24 h. Cells were then exposed to metformin, OA-Met, pro-DHA, triptolide, HAOPs, HAOTs, DexOPTs and HAOPTs with various concentration gradients. After incubation for 24 h, MTT solution (20 μL, 5 mg/mL) was added directly, and cells were incubated for another 4 h. The supernatant in well plates was substituted by DMSO (150 μL) to dissolve formazan crystals and the well plates were vibrated for 10 min. At last, well plates were subjected to microplate reader (Thermo Multiskan MK3, USA) for cell viability analysis at a wavelength of 570 nm and IC_50_ calculation.

The CI of OA-Met, pro-DHA or triptolide in micelles could be calculated by IC_50_ values based on Chou-Talalay theory and Eq. 3 [38, 39]:

3 $${\text{CI}} = \frac{{{\text{IC}}_{{50}} {\mkern 1mu} {\text{of}}{\mkern 1mu} {\text{drug}}{\mkern 1mu} \,{\text{A}}{\mkern 1mu} \,{\text{in}}\,{\mkern 1mu} {\text{combination}}\,{\mkern 1mu} {\text{therapy}}}}{{{\text{IC}}_{{50}} {\mkern 1mu} {\text{of}}\,{\mkern 1mu} {\text{drug}}{\mkern 1mu} \,{\text{A}}\,{\mkern 1mu} {\text{alone}}}} + \frac{{{\text{IC}}_{{50}} {\mkern 1mu} {\text{of}}{\mkern 1mu} {\text{drug}}\,{\text{B}}\,{\mkern 1mu} {\text{in}}\,{\mkern 1mu} {\text{combination}}{\mkern 1mu} \,{\text{therapy}}}}{{{\text{IC}}_{{50}} \,{\text{of}}{\mkern 1mu} {\text{drug}}{\mkern 1mu} \,{\text{B}}{\mkern 1mu} \,{\text{alone}}}} + \ldots$$

### Mammosphere formation assay and mammosphere formation efficacy (MSFE) calculation

The mammosphere formation assay was performed as follows. Briefly, 4T1 cells were seeded into ultra-low attachment 24-well plates (2 × 10^4^ cells/cm^2^) with DMEM/F12 culture medium containing B-27 Supplement (1 ×), EGF (20 ng/mL), bFGF (20 ng/mL), 100 mg/mL streptomycin and 100 U/mL penicillin (n = 3). Meanwhile, cells were treated with PBS buffer, metformin, OA-Met, pro-DHA, triptolide, HAOPs, HAOTs, DexOPTs and HAOPTs (10 ng/mL for triptolide). After 5 days of incubation for sphere formation, spheres were subjected to an inverted microscope (Leica DMI 4000B, Germany) for imaging and counting, and MSFE was calculated by following Eq. 4:

4 $${\text{MSFE}}\left( \% \right) = \frac{{{\text{number of spheres}}\,({\text{diameter greater than 50 }} \mu {\text{m}})}}{{\text{number of cells seeded }}} \times 100\%$$

### Flow cytometry assay for analysis of cancer stem cells in vitro

The phenotype of breast cancer stem cells (CD44^+^/CD24^−/low^) was identified by flow cytometry. After the mammosphere formation assay (n = 3), the cell suspensions were centrifuged at 800 rpm for 3 min to collect the spheres. Spheres were then treated with trypsin–EDTA (0.05%) for 3 min and centrifuged at 800 rpm for 3 min instantly. Additionally, single cells were collected, washed with PBS buffer (1 mL) and centrifuged at 1700 rpm for 5 min. After washing with PBS buffer for another two times, cells were stained with PE labeled anti-CD24 antibodies and FITC labeled anti-CD44 antibodies simultaneously away from light at 4 °C for 30 min. Finally, the stained cells were washed for three times and resuspended in PBS buffer (200 μL) for flow cytometric analysis by a BD FACScan (BD FACSAria II).

### Macrophages polarization assay in vitro

To investigate the ability of HAOPTs in the inhibition of M2 macrophage polarization, RAW264.7 cells were seeded into 6-well plates at the cell density of 1 × 10^5^ cells per well (n = 3). Macrophages were then stimulated with IL-13 (10 ng/ml) for 24 h followed by treating with PBS buffer, metformin, OA-Met, pro-DHA, triptolide, HAOPs, HAOTs, DexOPTs and HAOPTs (10 ng/mL for triptolide). After incubation for 24 h, cells were washed with PBS buffer for three times and collected by centrifuging at 1000 rpm for 4 min. Macrophages were then stained with PE labeled anti-CD206 antibodies in the dark at 4 °C for 30 min. At last, the stained cells were washed for three times and CD206-positive M2 macrophages were detected by flow cytometry (BD FACSAria II).

### Tumor cell invasion assay

The tumor cell invasion assay was performed through Transwell systems. Transwell membranes (8 μm pore size, Corning Costar Co., Cambridge, MA, USA) were coated with Matrigel and incubated at 37 °C for 4 h. The reconstituted Matrigel coatings were washed with RPMI 1640 twice and soaked for 30 min in RPMI 1640. 4T1 cells were suspended in RPMI 1640 containing 1% FBS (1 × 10^6^ cells/mL). The cell suspensions (100 μL) were seeded into the upper wells of coated Transwells and treated with PBS buffer, metformin, OA-Met, pro-DHA, triptolide, HAOPs, HAOTs, DexOPTs and HAOPTs (10 ng/mL for triptolide, final volume was 200 μL). Lower wells of the transwells were filled with RPMI 1640 containing 10% FBS, and PBS buffer, metformin, OA-Met, pro-DHA, triptolide, HAOPs, HAOTs, DexOPTs and HAOPTs (10 ng/mL for triptolide) were added to the lower wells (final volume was 500 μL). After incubation for 24 h, membranes coated with Matrigel were fixed with methanol at − 20 °C for 15 min, swabbed with a cotton swab and washed with PBS buffer for three times. Once the membranes were died, cells on the membranes were stained with crystal violet solution (500 μL) at 37 °C for 30 min. The transwells were then washed with PBS buffer and imaged under an inverted fluorescence microscope (Leica DMI 4000B, Germany). Afterward, the transwells were soaked in 33% acetic acid solution (500 μL) for 10 min, and the number of invasive cells was determined through the absorbance of crystal violet recorded by a microplate reader (Thermo Multiskan MK3, USA) at a wavelength of 570 nm.

### Immunohistochemical staining for orthotopic tumor sections

4T1 cells were inoculated into one mammary fat pad of Balb/c mice. On the sixth day after tumor inoculation, the mice were divided into nine groups randomly (n = 4) and infused with saline, metformin, OA-Met, pro-DHA, triptolide, HAOPs, HAOTs, DexOPTs and HAOPTs (0.7 mg/kg for triptolide) one time every two days via tail veins. Two days after the last injection (day 16), mice were euthanized and perfused with saline and 4% PFA, and tumors were harvested, fixed and embedded in paraffin. Moreover, the sections of tumors were applied for anti-mouse p-AMPK, Histone H3 (acetyl K9), E-cadherin, MMP-9, Collagen I antibodies and HRP double staining. The stained sections were finally subjected to an inverted fluorescence microscope (Leica DMI 4000B, Germany) for photographing and quantitative analysis was completed by using ImageJ 1.46 version program.

### Therapeutic efficacies of HAOPTs in orthotopic breast tumor mice

We adopted 4T1-Luc cell-derived orthotopic breast tumor mice to demonstrate the inhibitory effects of orthotopic breast tumor growth and spontaneous metastasis by HAOPTs. Briefly, 4T1-Luc cells were inoculated into one mammary fat pad of Balb/c mice. Once the volume of orthotopic tumors reached about 100 mm^3^, the mice were divided into eight groups randomly (n = 10) and intravenously injected with saline, OA-Met, pro-DHA, triptolide, HAOPs, HAOTs, DexOPTs and HAOPTs (0.7 mg/kg for triptolide) once every three days. The orthotopic tumor volumes were measured by a Vernier caliper during the therapeutic process. After all injections (day 27), five random mice from each group were euthanized and perfused with saline, and major organs (heart, liver, spleen, lung and kidney) and tumor tissues were collected. Additionally, tumors were weighed, fixed and embedded in paraffin, and tumor sections were stained with hematoxylin & eosin (H&E) or in situ cell death detection kit (TUNEL assay) followed by analysis of necrosis or apoptosis under an inverted fluorescence microscope (Leica DMI 4000B, Germany).

Furthermore, the metastasis suppression efficacy was evaluated by following methods. Harvested lung tissues were instantly soaked in D-luciferin solution (0.5 mg/mL) for 5 min, and then detected by an in vivo IVIS spectrum imaging system to record bioluminescence images and bioluminescent quantitative data. Besides, the rest five mice in each group were performed for the survival study.

### Toxicity evaluation of HAOPTs

In order to verify the safety of HAOPTs, the weight of mice was examined every three days during the above therapy. After all treatments (day 27), harvested major organs (heart, liver, spleen, lung and kidney) were served for H&E staining and imaging. Aspartate aminotransferase (AST), alanine aminotransferase (ALT) and alkaline phosphatase (ALP) levels in the serum were detected.

### Statistical analysis

All data were displayed as the mean ± standard deviation (SD). Student’s t-test was served for a difference between two groups, and the comparisons among multiple groups were analyzed by one-way ANOVA in Graphpad Prism 6.02. Statistical significance was presented with *P* value lower than 0.05.

## Results and discussions

### Synthesis and Characterization of pro-DHA

The HDAC inhibitor pro-DHA is a compound with the conjugation between propofol and DHA [40, 41]. Pro-DHA could be synthesized by a typical esterification reaction [41, 42]. The carboxyl group of DHA was activated under a catalytic condition of DCC/DMAP and then conjugated to the phenolic hydroxyl group of propofol. After purification by column chromatography, the HDAC inhibitor pro-DHA could be attained (Additional file 1: Scheme S1). The structure of pro-DHA was then verified by ^1^H-NMR (600 MHz) and ESI–MS. In ^1^H-NMR spectrum of pro-DHA, the phenolic hydroxyl peak of propofol at the δ value of 4.8 ppm disappeared. Moreover, hydrogen peak signals of carbons in propofol and DHA could be found simultaneously in the product (Additional file 1: Fig. S1A–C). Besides, two mass-to-charge ratios (m/z) of pro-DHA obtained by ESI–MS were 489.4 [M + H^+^] and 511.2 [M + Na^+^], respectively (Additional file 1: Fig. S1D). The results of ^1^H-NMR and ESI–MS demonstrated successful synthesis of pro-DHA.

### Preparation and Characterization of HAOPTs

**Fig. 2:**
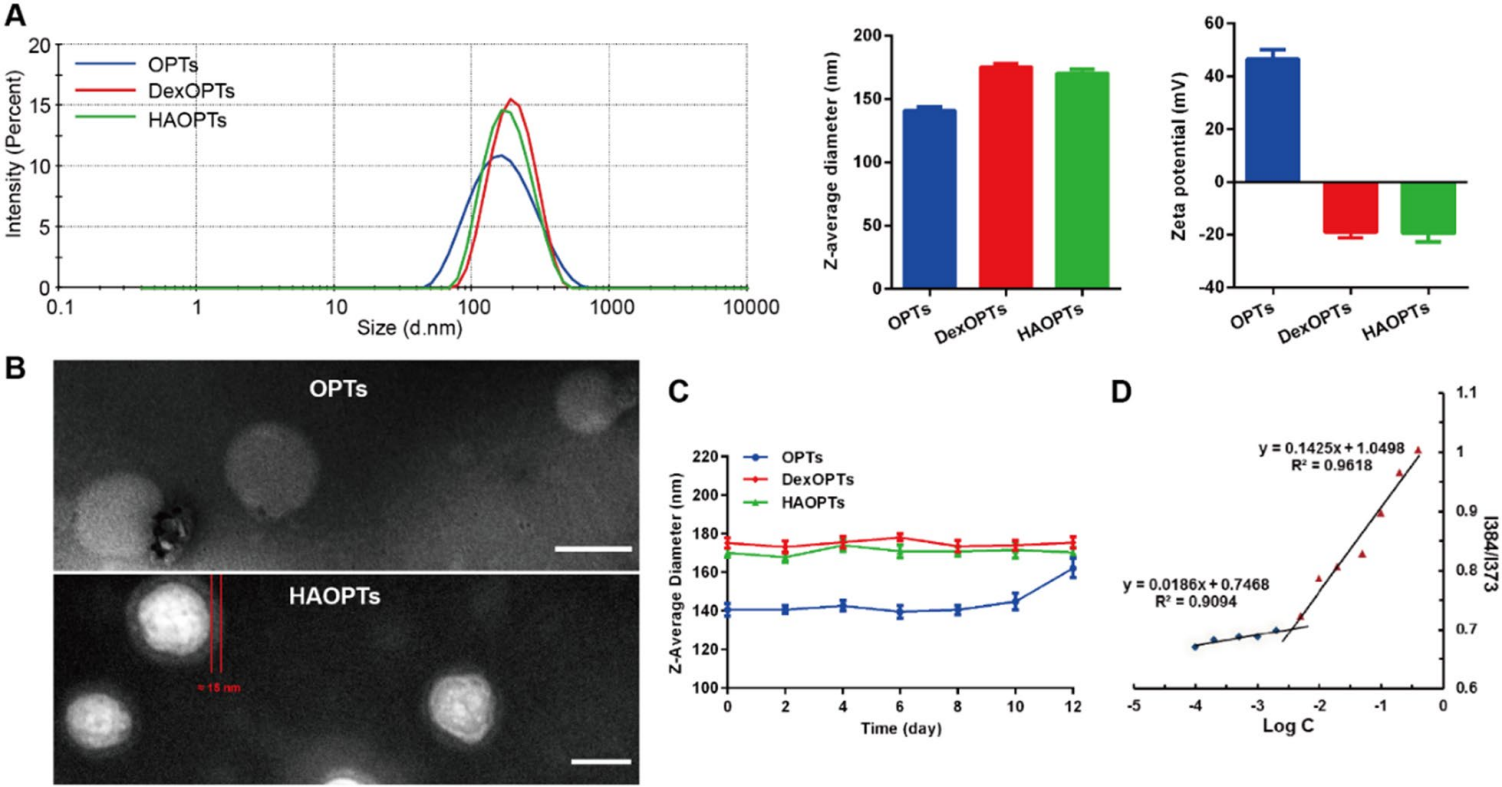
Characterization of HAOPTs. **A** Size distribution, z-average diameter and surface zeta potential of OPTs, DexOPTs and HAOPTs. Data were represented as mean ± SD (n = 3). **B** TEM images of OPTs and HAOPTs. Scale bar, 100 nm. **C** Z-average diameter alterations of different micelles (OPTs, DexOPTs and HAOPTs) with incubation in PBS at 4 °C for 12 days. Data were represented as mean ± SD (n = 3). **D** Plots of the fluorescence intensity ratio of 384 nm and 373 nm to the logarithm concentration of HAOPTs

OA-Met, a novel amphiphilic metformin derivative designed and synthesized by our research group [43], was developed by the conjugation of hydrophilic biguanide group in metformin and a hydrophobic carbon chain of oleic acid (Fig. 1A). OA-Met could form micelles in water, and only a compound with a long polyunsaturated carbon chain could be loaded in the hydrophobic cores to form hybrid micelles effectively [43]. Pro-DHA that possesses a long unsaturated carbon chain might be suitable for the hybrid with OA-Met, which was possibly attributable to the lipophilicity and π-π interaction between pro-DHA and the aliphatic chain of OA-Met [44, 45]. Triptolide was then loaded in the hydrophobic core of hybrid micelles to obtain OPTs, and OPTs finally adsorbed an anionic polysaccharide HA on their surfaces through electrostatic interaction to prepare HAOPTs. Moreover, dextran sulfate-modified OPTs (DexOPTs) were attained by the adsorption of dextran sulfate onto surfaces of OPTs. Based on positively charged biguanide groups, unmodified OPTs probably exhibited positive zeta potential, and adsorbed polysaccharide (dextran sulfate or HA) could shield positive charges on OPTs. OPTs and DexOPTs were considered as non-targeting micelles for control evaluations. The z-average diameter of OPTs, DexOPTs and HAOPTs was 140.7 ± 3.2 nm, 175.3 ± 2.6 nm and 170.2 ± 2.4 nm, respectively, and relevant PDI were 0.210 ± 0.014, 0.124 ± 0.019 and 0.124 ± 0.016, respectively. Zeta potentials of OPTs, DexOPTs and HAOPTs were 46.4 ± 3.6 mV, -19.1 ± 2.2 mV and -19.6 ± 3.2 mV, respectively (Fig. 2A, Additional file 1: Table S1). Additionally, compared with the morphology of OPTs (Fig. 2B, Additional file 1: Fig. S2), HAOPTs displayed a polysaccharide layer (about 15 nm) analyzed by a TEM. Surface decoration of HA on OPTs was ensured by size increase (about 30 nm), charge reversal (positive to negative charge) and adsorbed layer in the TEM image of HAOPTs. The EE of pro-DHA and triptolide in HAOPTs was 69.1 ± 2.9% and 86.5 ± 2.0%, and the LC was 9.4 ± 0.4% and 2.45 ± 0.20%, respectively (Additional file 1: Table S2 and Table S3), indicating that pro-DHA was mixed with OA-Met to form hybrid micelles entrapping triptolide efficiently. To obtain OPTs and subsequent HAOPTs with ideal z-average diameter and PDI, optimized mass ratio of OA-Met, pro-DHA and triptolide was necessary during micelle preparation (Micelle 5 in Additional file 1: Table S4), and the formation of ultimate micelles might profit from hydrophobic effects and conjugative effects among OA-Met, pro-DHA and triptolide. Furthermore, outstanding stability of polysaccharide modified OPTs (DexOPTs and HAOPTs) was observed in PBS (pH 7.4) at 4 °C for more than 12 days while OPTs displayed evident increase of diameter from day 8 (Fig. 2C). The CMC of HAOPTs was 3.59 μg/mL (Fig. 2D), exhibiting excellent anti-dilution ability in blood [46].

### Cellular uptake of HAOPTs in breast tumor cells

**Fig. 3:**
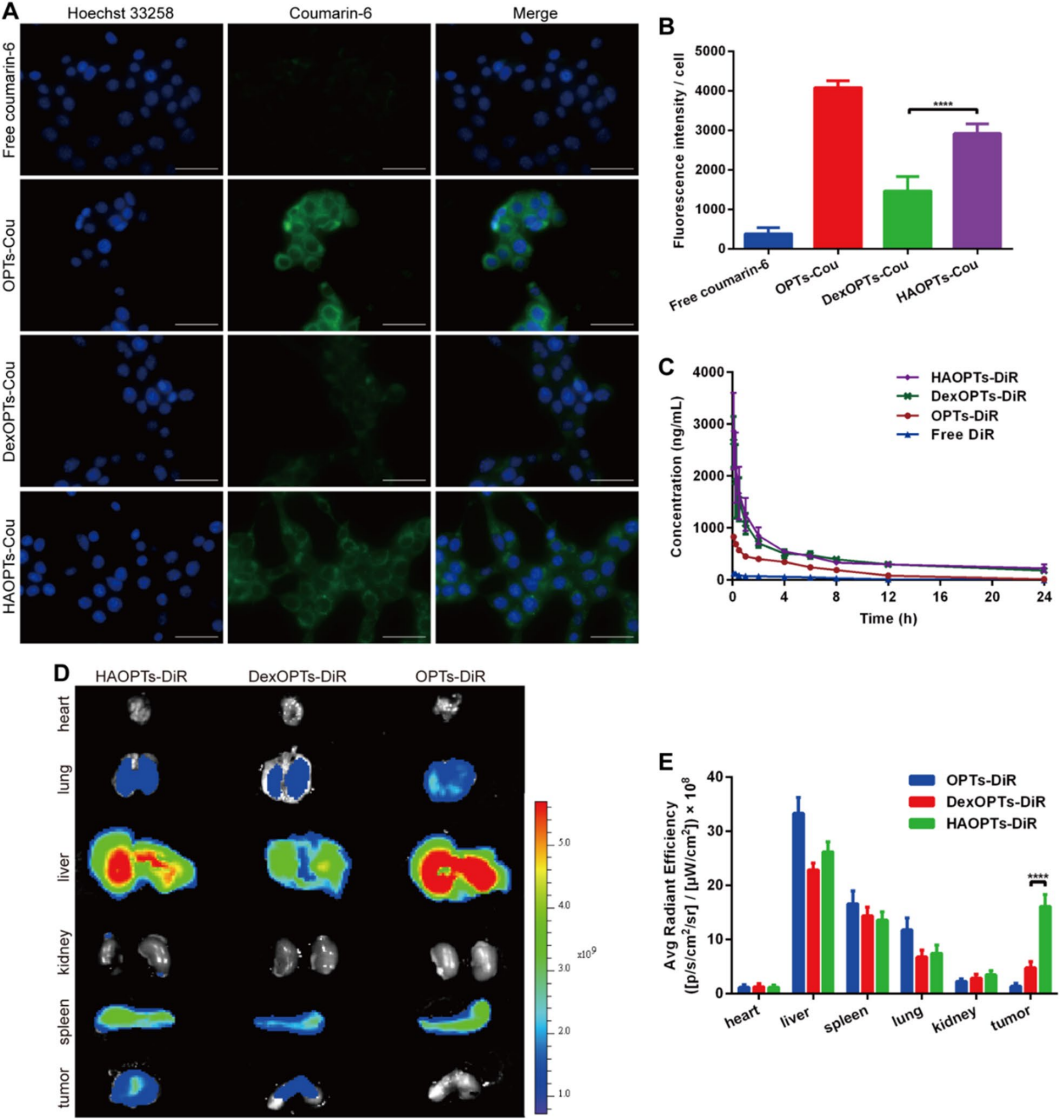
HAOPTs targeted orthotopic breast tumor tissues. **A** Fluorescent images of the cellular uptake of free coumarin-6, OPTs-Cou, DexOPTs-Cou and HAOPTs-Cou in 4T1 tumor cells. Blue, Hoechst 33,258; green, coumarin-6. Scale bar, 50 µm. **B** Quantitative analysis of the cellular uptake in 4T1 cells. **C** Plasma DiR concentration–time curves after intravenous injection of free DiR, OPTs-DiR, DexOPTs-DiR and HAOPTs-DiR in S-D rats. Data were represented as mean ± SD (n = 3), ****P < 0.0001. **D** Representative ex vivo fluorescence images of hearts, lungs, livers, kidneys, spleens and tumors after 24 h. **E** Semi-quantitative analysis of the fluorescence intensity of ex vivo tumors and other organs. Data were represented as mean ± SD (n = 3), ****P < 0.0001

The orthotopic tumor targeting efficacy of HAOPTs was firstly assessed in murine breast tumor cell lines 4T1 cells [47]. On account of the interaction between positively charged coumarin-6 labeled OPTs (OPTs-Cou) and negatively charged cell membranes, the fluorescence of OPTs-Cou in 4T1 cells was the brightest among all groups. The accumulation of coumarin-6 labeled HAOPTs (HAOPTs-Cou) was significantly stronger than that of coumarin-6 labeled DexOPTs (DexOPTs-Cou) (*P* < 0.0001), and there was almost no cellular uptake of free coumarin-6 in 4T1 cells as predicted (Fig. 3A, B). Due to the overexpression of CD44 on the surface of tumor cells [36], the above results suggested that high affinity between HA and CD44 had a crucial role in the targeting effect of HAOPTs to 4T1 tumor cells [36, 48].

### Polysaccharide adsorption enhanced pharmacokinetic properties of OPTs

The targeting efficacy of HAOPTs should be investigated in vivo following the cellular uptake study. First of all, the blood circulation profile was evaluated through the administration of DiR labeled micelles (OPTs-DiR, DexOPTs-DiR and HAOPTs-DiR) [49, 50]. The concentrations of OPTs-DiR, DexOPTs-DiR and HAOPTs-DiR in plasma were obtained by DiR fluorescence measurement (Fig. 3C) followed by the calculation of key pharmacokinetic parameters (Additional file 1: Table S5) such as area under curve (AUC_0-24 h_), blood circulation half-life (t_1/2_), clearance (CL) and mean residence time (MRT_0-24 h_). The AUC_0-24 h_ of DexOPTs-DiR and HAOPTs-DiR was 2.55- and 2.70-fold higher than that of OPTs-DiR, respectively. Moreover, both polysaccharide-decorated OPTs owned longer t_1/2_ than OPTs (14.65 h, 13.12 h and 4.09 h for HAOPTs-DiR, DexOPTs-DiR and OPTs-DiR, respectively, *P* < 0.01). The clearance of DexOPTs-DiR and HAOPTs-DiR was obviously slower than that of OPTs-DiR (*P* < 0.001 for CL), and the MRT_0-24 h_ of DexOPTs-DiR and HAOPTs-DiR was found to be 1.38- and 1.41-fold longer than that of OPTs-DiR, respectively. Compared with DiR labeled micelles, free DiR was eliminated the most quickly as expected, and positively charged OPTs-DiR were eliminated faster than the polysaccharide-modified micelles (DexOPTs-DiR and HAOPTs-DiR) probably due to the recognition through mononuclear phagocyte system (MPS) [51, 52]. Therefore, the results demonstrated that polysaccharide (HA and dextran sulfate) adsorption enhanced the pharmacokinetic properties of OPTs.

### HAOPTs targeted orthotopic breast tumors in vivo

In vivo targeting ability of HAOPTs to orthotopic tumors should be studied based on excellent cellular uptake and pharmacokinetic properties of HAOPTs. The biodistribution assay was performed in 4T1 cell-derived orthotopic tumor mice by the intravenous injection of DiR labeled micelles. As shown in Additional file 1: Fig. S3, the accumulation of HAOPTs-DiR in tumor tissues increased gradually and was higher than that of OPTs-DiR and DexOPTs-DiR at predesigned time points. After 24 h, HAOPTs-DiR exhibited the brightest fluorescence in orthotopic tumors according to the semi-quantitative analysis of ex vivo organs as well (*P* < 0.0001, Fig. 3D, E). Besides, micelles could be partially cleared from the blood via the accumulation in the organs of MPS such as liver and spleen (Fig. 3D) [53], which conformed to the pharmacokinetic results (Fig. 3C). In brief, compared with OPTs and DexOPTs, the targeting effect of HAOPTs to orthotopic tumors was owing to the long circulation of HAOPTs and the high affinity of HA for CD44 [54].

### HAOPTs showed cytotoxicity and synergistic effects on tumor cells

Since inspiring targeting efficacies of HAOPTs were determined, we started to evaluate the therapeutic mechanisms of HAOPTs. Primarily, the inhibition of proliferation against 4T1 cells should be assessed through a MTT assay. The efficacy of combinations needed to be examined objectively with reasonably designed controls which would offer necessary evidences for any enhancement outcomes. Thus, HA modified OA-Met and pro-DHA hybrid micelles (HAOPs) and HA modified OA-Met micelles loading triptolide (HAOTs) were prepared based on the preparation method of HAOPTs. HAOPs and HAOTs were studied as control groups for the effect of missing one drug in HAOPTs. According to the cell viability curves, OA-Met possessed an IC_50_ value of 7.88 μg/mL on 4T1 cells (Additional file 1: Fig. S4A). Additionally, the IC_50_ value of pro-DHA was 5.01 μg/mL, and IC_50_ values of pro-DHA and OA-Met could decrease to 1.70 μg/mL and 3.25 μg/mL in HAOPs (Additional file 1: Fig. S4B). For the triptolide-associated groups (Additional file 1: Fig. S4C), free triptolide owned IC_50_ value with nanogram level (47.96 ng/mL) and exhibited stronger cytotoxicity than OA-Met and pro-DHA. When combined with OA-Met in HAOTs, the cytotoxicity of triptolide (IC_50_ = 37.05 ng/mL) was 1.29-fold higher than that of free triptolide. Triptolide in three drug combined micelles (DexOPTs and HAOPTs) displayed IC_50_ values of 27.62 ng/mL and 18.8 ng/mL, which were 1.74- and 2.55-fold lower than that of free triptolide, respectively. The toxicity of HAOPTs was much stronger than that of DexOPTs probably due to better uptake of HAOPTs in tumor cells. Collectively, the above data indicated that triptolide was the most potent drug in tumor cell toxicity, and the combination of triptolide with OA-Met (HAOTs) or both OA-Met and pro-DHA (DexOPTs and HAOPTs) enhanced the cytotoxicity of triptolide on tumor cells efficiently.

To investigate the combination effects of OA-Met, pro-DHA and triptolide, we calculated CI by IC_50_ values [38, 39]. In micelle HAOTs, the CI value of triptolide and OA-Met was 0.834. Furthermore, the CI for the combination of OA-Met, pro-DHA and triptolide was 0.443 in HAOPTs. We noticed that all above CI values were less than 1 (CI < 1), suggesting the combinations of multi-drugs in HAOTs and HAOPTs were potently synergistic. Particularly, CI value of OA-Met, pro-DHA and triptolide in HAOPTs was lower than HAOTs, indicating that the combination of three drugs showed a stronger synergistic effect than the two-drug combination.

### HAOPTs inhibited mammosphere formation and reduced cancer stem cell subpopulations with CD44^+^/CD24^−/low^ Phenotype

**Fig. 4:**
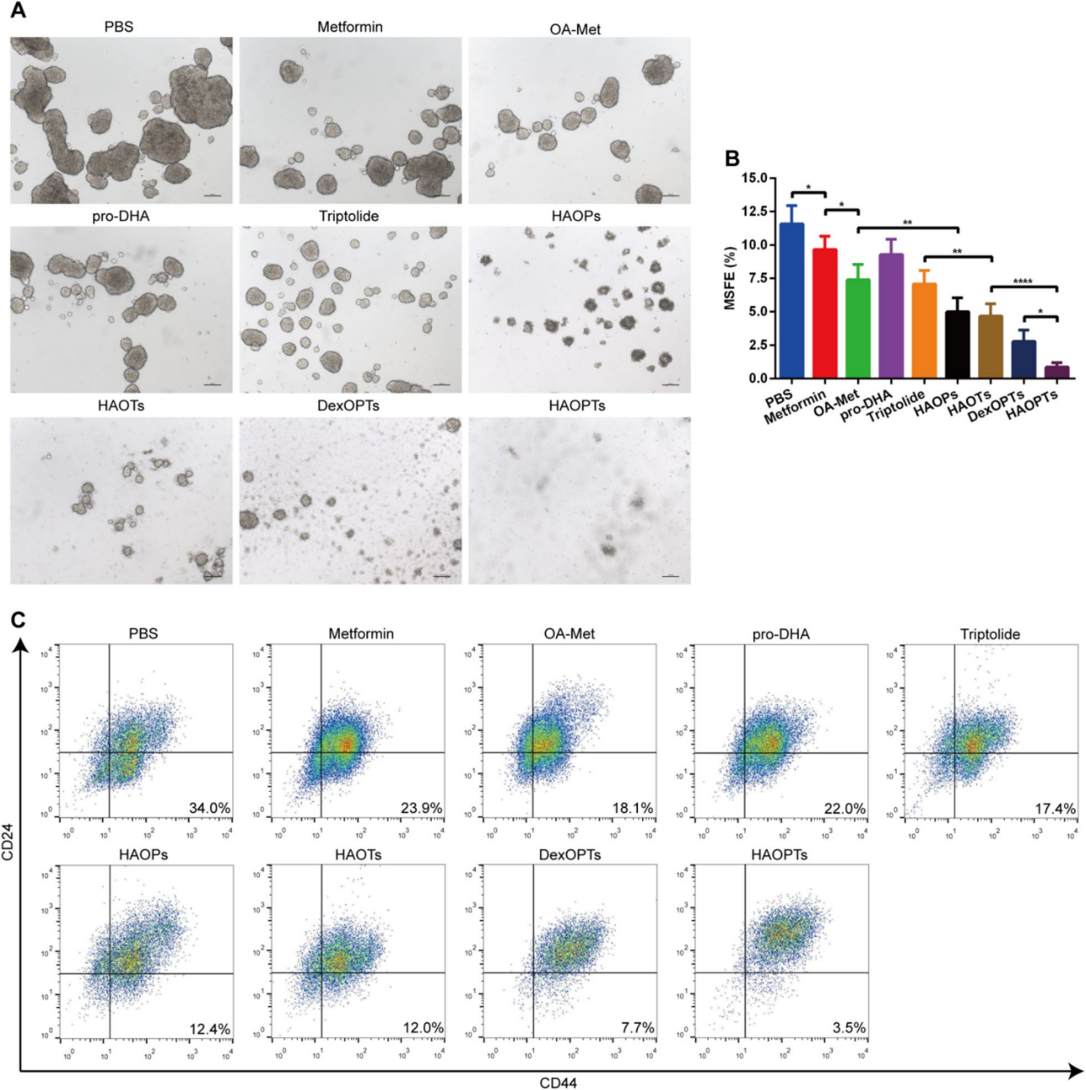
Inhibitory effects on cancer stem cell-like 4T1 cells. **A** Representative morphology of tumor spheres formed by cancer stem cell-like 4T1 cells incubated with free metformin, free OA-Met, free pro-DHA, free triptolide, HAOPs, HAOTs, DexOPTs and HAOPTs. Scale bar, 100 µm. **B** Quantitative analysis of the MSFE on cancer stem cell-like 4T1 cells. Data were represented as mean ± SD (n = 3), *P < 0.05, **P < 0.01, ****P < 0.0001. **C** Flow cytometry analysis of CD44^+^/CD24^−/low^ subpopulation in cancer stem cell-like 4T1 cells

Tumors are widely recognized as a stem cell-related disease. The EMT process is associated with CSC production, a type of cells owning survival, differentiation and self-renewal abilities, and CSCs act vital roles in tumorigenesis, metastasis and recurrence [55]. It is suggested that specific inhibition of CSCs is an effective strategy for treating tumors, metastasis and recurrence [56, 57]. For above reasons, we adopted mammosphere formation assay, a well-established method for evaluating the formation of CSC-like cells, to investigate the inhibitory effects of HAOPTs on the stemness of tumor cells. Tumor cells were stimulated to form spheres in a special serum-free stem cell-inducing medium, and the inhibition of stemness was examined by sphere size, morphology and MSFE [58, 59]. As shown in Fig. 4A, B, the untreated PBS group formed obviously large tumor spheres with the highest MSFE among all groups. Compared with PBS group, tumor spheres were smaller and more dispersed in metformin, OA-Met, pro-DHA and triptolide groups, and the MSFE also significantly decreased (*P* < 0.05). The suppression effect of OA-Met was stronger than metformin (*P* < 0.05) probably due to the improved hydrophobicity. In addition, the tumor sphere diameters and MSFEs of HAOPs, HAOTs, DexOPTs and HAOPTs groups with drug combination were further reduced (*P* < 0.01), and the visible scattered single cells were more clear in micelle groups. On account of the combination of three drugs (OA-Met, pro-DHA and triptolide), HAOPTs displayed prominently better inhibitory effects of CSCs than HAOPs and HAOTs (*P* < 0.0001). Moreover, the suppression of HAOPTs was stronger than that of DexOPTs (*P* < 0.05) in keeping with the superb cellular uptake of HAOPTs.

In order to further confirm inhibitory effects of CSCs, we continued to test the phenotype of cells after the mammosphere formation assay. Many literatures have reported that CSCs express particular marker antigens in tumor tissues, and different tumors can develop various CSC phenotypes [60]. In breast tumors, CD44^+^/CD24^−/low^ is the specific cell phenotype of breast CSCs, so we determined the proportion of CSC subsets (CD44^+^/CD24^−/low^) in all administration groups by flow cytometry [61, 62]. After mammosphere count and morphology observation, the proportion of CSC subpopulations in the PBS group was the highest among all groups (about 34%, Fig. 4C, Additional file 1: Fig. S5). CSC subsets in metformin, OA-Met, pro-DHA and triptolide groups were fewer than PBS group (*P* < 0.0001), and the suppression of OA-Met was stronger than metformin as well (*P* < 0.01). Additionally, the percentage of CSCs in drug combination micelles (HAOPs, HAOTs, DexOPTs and HAOPTs) decreased significantly (*P* < 0.01), and HAOPTs possessed the most inhibition of CSCs among all micelle groups (*P* < 0.0001 for HAOPs and HAOTs,* P* < 0.05 for DexOPTs, Fig. 4C, Additional file 1: Fig. S5). Overall, the investigations of CSCs confirmed the prominent efficacies of HAOPTs in CSC suppression, improving the possibility of metastasis prevention and tumor suppression.

### HAOPTs suppressed M2 macrophage polarization

**Fig. 5:**
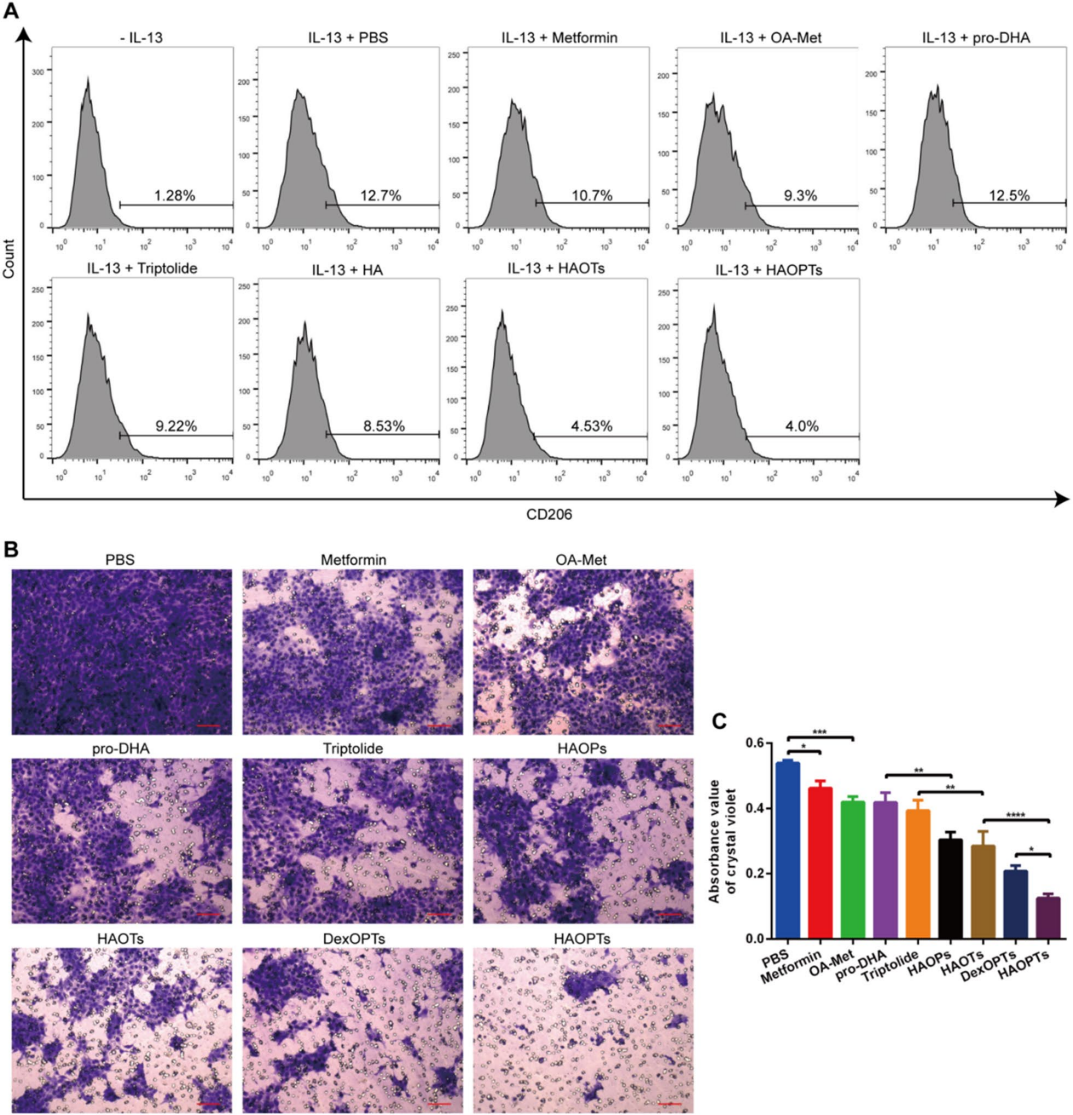
HAOPTs suppressed the polarization of M2 macrophages and tumor cell invasion in vitro. **A** Flow cytometry analysis of CD206 on macrophages in different treatment groups. **B** Images of invasive tumor cells stained by crystal violet in matrigel invasion assay. Scale bar, 100 µm. **C** Quantitative analysis of tumor cell invasion. Data were represented as mean ± SD (n = 3), *P < 0.05, **P < 0.01, ***P < 0.001, ****P < 0.0001

Monocytes in peripheral blood infiltrate into tumor tissues for the formation of tumor-associated macrophages. The initial macrophages can polarize to M2 phenotype that promote tumor growth by the stimulation of inflamed tumor microenvironments [13, 63, 64]. Meanwhile, M2 macrophages can also secrete cytokines to promote EMT processes and tumor metastasis, hence the impacts of HAOPTs should be assessed in M2 macrophage polarization [18, 65]. IL-13 has been reported to prompt M2 polarization of macrophages [66], and CD206 is a macrophage mannose receptor, which can be identified as a marker antigen of M2 macrophages [63]. According to the flow cytometry results, the positive incidence of M2 macrophages increased from 1.28% to 12.7% under IL-13 stimulation (*P* < 0.0001). Metformin, OA-Met, triptolide (*P* < 0.05) and even HA (*P* < 0.001) significantly inhibited M2 polarization (Fig. 5A, Additional file 1: Fig. S6). HA can inhibit M2 polarization via the interaction with toll-like receptor (TLR) on macrophage membranes and the TLR-dependent pathway [63]. Free metformin (or metformin derivative OA-Met) and triptolide can suppress M2 polarization [67, 68], while pro-DHA presented no reduction in M2 polarization possibly because of the induction of M2 polarization by HDAC inhibitory functions [69]. The combination of HA, OA-Met and triptolide in HAOTs further reduced M2 macrophages compared with free drug groups (*P* < 0.001), and similar inhibitory effects were observed in HAOPTs groups in spite of the addition of pro-DHA in HAOTs. After M2 macrophage inhibition via the interaction between HA and TLR, HA modification on HAOTs and HAOPTs can promote the internalization into macrophage [70], leading to further suppression effects from OA-Met and triptolide. In conclusion, we demonstrated that M2 macrophages development could be prevented by HAOPTs effectively.

### HAOPTs inhibited tumor cell invasion

Tumor metastasis is exceedingly dependent on the invasive ability of tumor cells. Orthotopic tumor cells can obtain invasive abilities during EMT processes and migrate into the circulatory system through extracellular matrix (ECM) degradation. The degradation process is closely associated with the secretion of MMPs by tumor cells [7, 71]. Otherwise, Matrigel, a natural matrix extracted from mouse sarcoma, contains components such as type IV collagen, laminin and heparin sulfate. The membrane formed by Matrigel can mimic the ECM structure [72]. In this assay, we applied a layer of Matrigel on Transwell membranes to simulate ECM, and investigated whether tumor cells could pass through Matrigel layers under drug treatments. As presented in Fig. 5B, C, the absorbance of invasive cells stained by crystal violet in free drug groups was weaker than that in the PBS group (*P* < 0.05), and HAOPs, HAOTs, DexOPTs and HAOPTs further reduced invasive cells obviously (*P* < 0.01). In addition, HAOPTs blocked more cells from invasion than HAOPs and HAOTs (*P* < 0.0001), and the invasive cells in HAOPTs group were also greatly fewer than those in DexOPTs group (*P* < 0.05). These observations supported that, by restraining tumor cell invasion, HAOPTs might display efficient inhibitory activity against metastasis formation.

### HAOPTs up-regulated phospho-AMPK and exhibited HDAC inhibitory effects

**Fig. 6:**
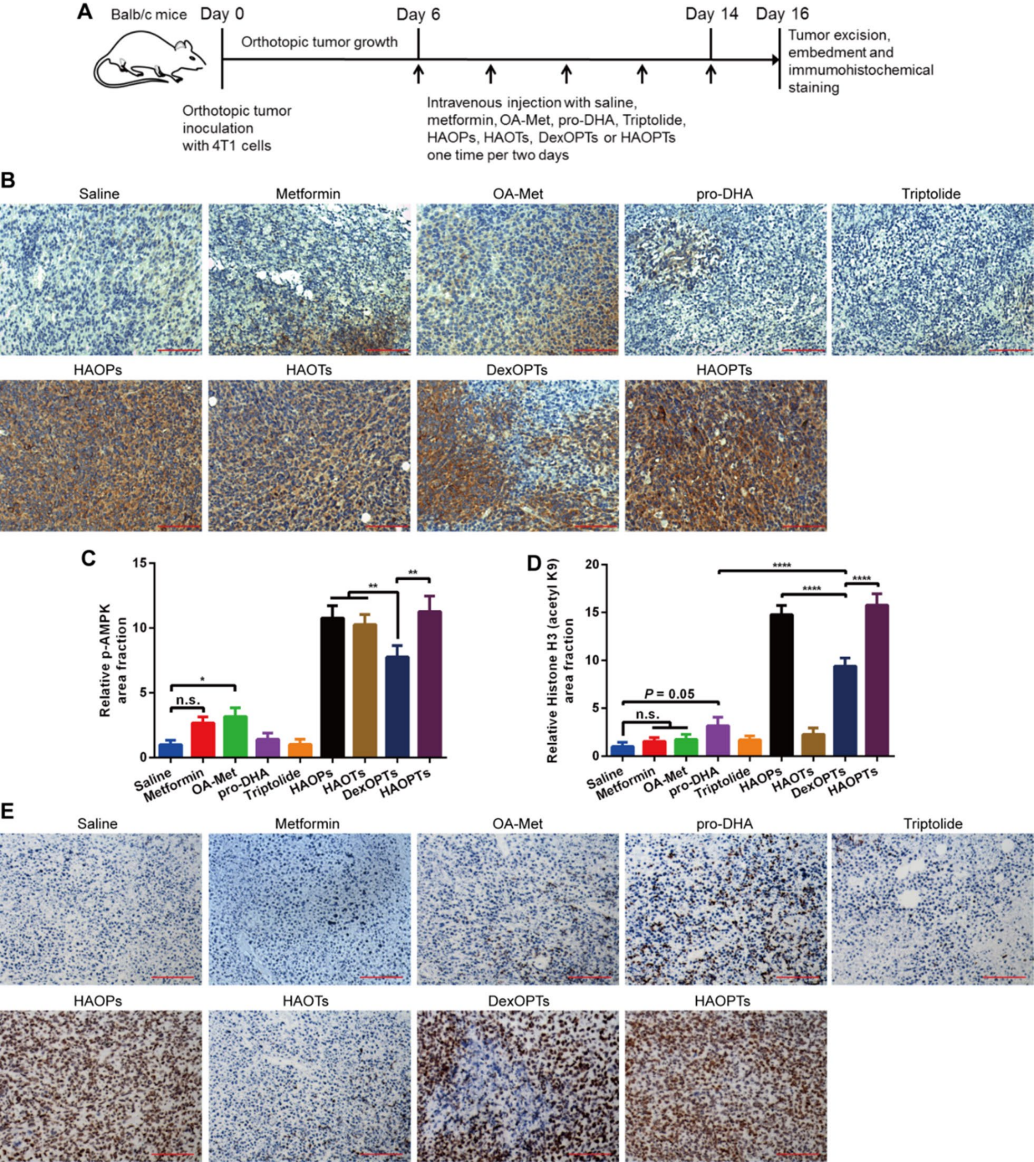
AMPK activation (AMPK phosphorylation) and HDAC inhibition activities in mice breast tumor tissues. **A** Timeline and treatment schedule for immunohistochemical staining of tumor tissues treated with free metformin, free OA-Met, free pro-DHA, free triptolide, HAOPs, HAOTs, DexOPTs and HAOPTs. **B** Images of breast tumor tissues stained by immunohistochemical staining with p-AMPK (brown). Scale bar, 100 µm. Quantitative analysis of **C** p-AMPK positive area and **D** Histone H3 (acetyl K9) positive area. Data were represented as mean ± SD (n = 4), *P < 0.05, **P < 0.01, ****P < 0.0001. **E** Images of breast tumor tissues stained by immunohistochemical staining with Histone H3 (acetyl K9) (brown). Scale bar, 100 µm

Metformin can present inhibitory effects against EMT through AMPK phosphorylation (p-AMPK) that suppress subsequent downstream inflammatory pathways. HAOPTs and other micelles were prepared on account of the metformin derivative OA-Met, thus we should examine whether OA-Met in micelles could activate AMPK by the degree of AMPK phosphorylation in immunohistochemical sections of orthotopic tumor tissues [73, 74]. After five doses of treatments (Fig. 6A), immunohistochemical analysis revealed that saline, pro-DHA and triptolide groups hardly expressed p-AMPK, whereas OA-Met could weakly activate AMPK with a small amount of p-AMPK expression (*P* = 0.0491) and metformin had weaker AMPK activation than OA-Met (Fig. 6B, C). Additionally, HAOPs, HAOTs, DexOPTs and HAOPTs were all prepared based on OA-Met and showed stronger effects on AMPK activation compared with metformin and OA-Met due to excessive clearance and tumor targeting deficiency of free drugs. The accumulation of p-AMPK in HAOPs, HAOTs and HAOPTs groups was higher than that in DexOPTs group (*P* < 0.01), which could be attributed to the long circulation and the tumor targeting efficacy of HA modified micelles (Fig. 6B, C). This study proved that OA-Met exhibited similar AMPK activation to metformin, providing a basis for following therapeutic effects of OA-Met.

HDAC can directly affects the transcription factors related to inflammation. HAOPTs comprised HDAC inhibitory agent pro-DHA, and the HDAC inhibitory efficiency of HAOPTs should be verified in tumor tissues. Since HDAC could catalyze the deacetylation of histones, we directly inspected the degree of Histone H3 deacetylation in tumor tissues for the effect of HAOPTs on HDAC inhibition [75–77]. According to immunohistochemical results (Fig. 6D, E), Histone H3 in saline group was almost deacetylated. Metformin, OA-Met and triptolide displayed no effect on HDAC, whereas pro-DHA could inhibit HDAC with a small quantity of acetylated Histone H3 expression (*P* = 0.05). In micelle groups, only HAOTs showed almost no accumulation of acetylated Histone H3 because of the absence of pro-DHA, and the tumor-targeted micelles HAOPs and HAOPTs were more potent than DexOPTs (*P* < 0.0001) in HDAC suppression. These results validated the essential role of pro-DHA in HAOPTs for the inhibition of HDAC.

### HAOPTs reversed abnormal expression of E-cadherin, MMP-9 and collagen? in tumor EMT processes

**Fig. 7:**
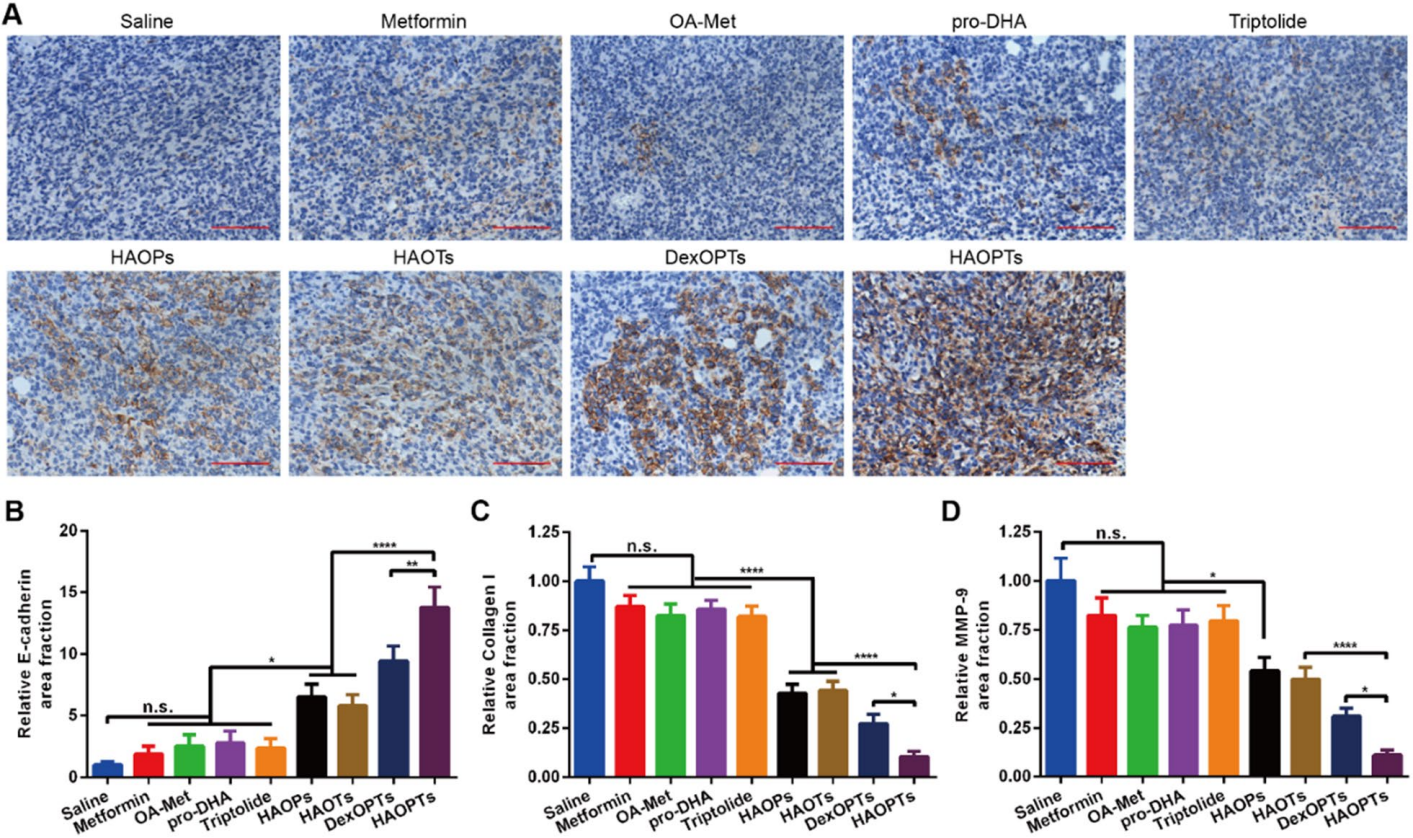
Modulation of E-cadherin expression, Collagen I accumulation and MMP-9 expression in mice breast tumor tissues. **A** Images of breast tumor tissues stained by immunohistochemical staining with E-cadherin (brown). Scale bar, 100 µm. Quantitative analysis of **B** E-cadherin positive area, **C** Collagen I positive area and **D** MMP-9 positive area. Data were represented as mean ± SD (n = 4), *P < 0.05, **P < 0.01, ****P < 0.0001

Aberrant expression of key proteins including E-cadherin, MMP-9 and Collagen I is strictly related to the development of EMT processes in tumor tissues. E-cadherin is an essential intercellular adhesion molecule inducing the establishment of stable junctions among epithelial cells**.** Loss of E-cadherin expression reduces the adhesion between tumor cells and promotes cell invasion, which is considered as one of the crucial markers of EMT processes [7, 78, 79]. Consistent with the researches of p-AMPK and Histone H3 (Fig. 6A), the exploration of E-cadherin was also achieved by immunohistochemical staining in tumor sections (Fig. 7A, B). E-cadherin was barely expressed in the saline group, and free drugs (metformin, OA-Met, pro-DHA and triptolide) slightly increased E-cadherin expression with no significant differences in contrast with the saline group. The inconspicuous difference was possibly attributed to targeting deficiency and quick clearance of free drugs in vivo. Owing to drug combination and long circulation, the accumulation of E-cadherin in HAOPs, HAOTs, DexOPTs and HAOPTs groups was higher than free drug groups (*P* < 0.05), and the promoting effect of HAOPTs with the combination of three drugs was more remarkable than HAOPs and HAOTs (*P* < 0.0001). In addition, tumor-targeted HAOPTs could realize better promotion compared with DexOPTs (*P* < 0.01).

Besides, tumor microenvironments stimulate fibroblasts to secrete ECM component Collagen I in tumor tissues. Collagen I accumulation can facilitate cytoskeletal remodeling of tumor cells and inhibit E-cadherin expression, inducing the EMT process cooperate to enhance cell migration ability [7, 80]. On the basis of the immunohistochemical results in Fig. 7C and Additional file 1: Fig. S7A, the Collagen I accumulation in tumors of untreated mice was the highest among all groups, while Collagen I expression in the free drug groups decreased slightly with weak inhibitory effects. HAOPs, HAOTs, DexOPTs and HAOPTs prominently reduced Collagen I accumulation (*P* < 0.0001), and HAOPTs achieved the most potent depression effect in contrast with HAOPs, HAOTs (*P* < 0.0001) and DexOPTs (*P* < 0.05).

Furthermore, MMP-9 is up-regulated by tumor cells in tumor tissues [71, 81]. Overexpressed MMP-9 can accelerate the invasion and migration of tumor cells into the circulatory system by degradation and remodeling of ECM. Additionally, MMP-9 accumulation can also damage E-cadherin, facilitating EMT processes of tumor cells [7, 71]. Immunohistochemical staining analysis of MMP-9 (Fig. 7D, Additional file 1: Fig. S7B) showed that MMP-9 was obviously expressed in tumors of saline treated mice. The expression of MMP-9 was slightly depressed by free drugs, and various micelles (HAOPs, HAOTs, DexOPTs and HAOPTs) could significantly suppress MMP-9 accumulation compared with free drug groups (*P* < 0.05). Consistent with the Collagen I decline, HAOPTs performed better inhibition than HAOPs, HAOTs (*P* < 0.0001) and DexOPTs (*P* < 0.05). In conclusion, the abnormal expression of E-cadherin, MMP-9 and Collagen I in tumors was amended effectively, revealing the prevention of EMT processes and prospective metastasis suppression ability of HAOPTs.

### HAOPTs repressed orthotopic tumor growth and metastasis establishment

**Fig. 8:**
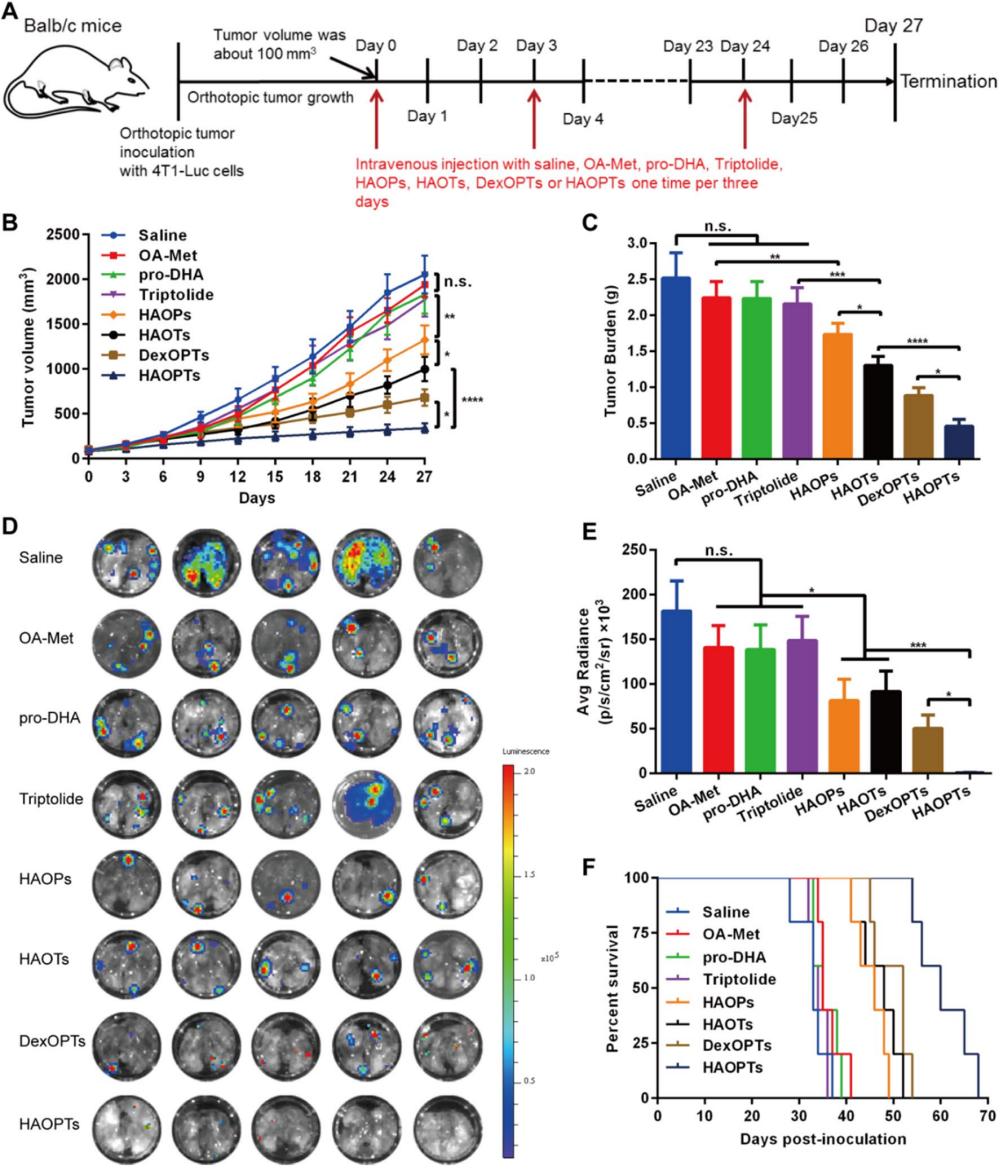
HAOPTs inhibited primary tumor growth and lung metastasis formation in orthotopic breast tumor mice. **A** Treatment schedule and timeline for treatments of free OA-Met, free pro-DHA, free triptolide, HAOPs (27.9 mg/kg), HAOTs (25.9 mg/kg), DexOPTs (30.2 mg/kg) and HAOPTs (28.6 mg/kg). **B** Primary tumor growth curves of mice in 27 days. **C** The analysis of ex vivo tumor weights after all treatments. Data were represented as mean ± SD (n = 5), *P < 0.05, **P < 0.01, ***P < 0.001, ****P < 0.0001. **D** Lung ex vivo images with bioluminescent signals after all treatments. **E** Lung ex vivo semi-quantitative analysis with bioluminescence. Data were represented as mean ± SD (n = 5), *P < 0.05, ***P < 0.001. **F** Kaplan–Meier survival analysis of mice

After examining the therapeutic mechanisms of HAOPTs in EMT processes or tumor development, we finally inspected whether HAOPTs could block metastasis establishment and suppress tumor growth simultaneously. On the basis of the mechanism studies, we hypothesized that HAOPTs might generate outstanding suppression in tumor growth mainly though cytotoxicity, CSC suppression and M2 macrophage inhibition. Thus, three days after the last treatment (day 27) in 4T1-Luc cell derived orthotopic breast tumor mice (Fig. 8A), tumor volumes and tumor burdens in free drug groups (OA-Met, pro-DHA and triptolide) were slightly less than the saline group owing to targeting absence and short circulation (Fig. 8B, C, Additional file 1: Fig. S8). The drug combination micelles HAOPs, HAOTs, DexOPTs and HAOPTs effectively reduced tumor volume (*P* < 0.01) and tumor burden (*P* < 0.01) in contrast with free drug groups, and HAOTs performed much stronger inhibition of tumor growth than HAOPs based on the nanogram level cytotoxicity of triptolide (*P* < 0.05). Moreover, HAOPTs displayed the least tumor burden (*P* < 0.05) and tumor volume (*P* < 0.05) attributed to the combination of three drugs and tumor targeting effect. Besides, the necrosis and apoptosis of tumor tissues were also investigated by H&E staining and TUNEL assay, respectively. There was almost no necrosis tissue in OA-Met and pro-DHA groups while the triptolide group showed a small necrotic area. HAOPs, HAOTs, DexOPTs, and HAOPTs resulted in more necrosis, and HAOPTs produced the largest necrotic area among all groups (Additional file 1: Fig. S9). Similarly, free drug groups exhibited a few apoptotic cells with no significant difference compared with the saline group. The area of apoptotic tissues in HAOPs, HAOTs, DexOPTs and HAOPTs groups was significantly extended (*P* < 0.001), and HAOPTs realized the best proapoptotic effect (*P* < 0.01, Additional file 1: Fig. S10). The TUNEL assay indicated that HAOPTs could also treat tumor growth by inducing apoptosis of tumor cells [82].

HAOPTs might display potential inhibitory efficacies of EMT via inflammation related CSC suppression, M2 macrophage inhibition, cell invasion reduction, AMPK activation, HDAC inhibition, up-regulation of E-cadherin and down-regulation of Collagen I and MMP-9. To verify the therapeutic effects of HAOPTs against metastasis by EMT inhibition, the lungs were harvested for IVIS bioluminescence detection. As shown in Fig. 8D, only three mice appeared weak lung metastasis in the HAOPTs group, whereas all mice from other groups established serious lung metastasis according to the lung bioluminescence. In addition, semi-quantitive analysis discovered that OA-Met, pro-DHA and triptolide could slightly inhibit lung metastasis (Fig. 8E). HAOPs, HAOTs, DexOPTs and HAOPTs were more effective than free drug groups (*P* < 0.05) as expected, and HAOPTs possessed the strongest metastasis prevention ability (*P* < 0.05). Otherwise, the survival research revealed that the HAOPTs group provided the longest medium survival time (60 days), while the medium survival time of saline, OA-Met, pro-DHA, triptolide, HAOPs, HAOTs and DexOPTs group was 33, 35, 35, 34, 46, 48 and 52 days, respectively (Fig. 8F). Overall, the comprehensive therapy of HAOPTs could be achieved by the simultaneous inhibition of tumor growth and metastasis formation, which prolonged the survival time.

### Safety of HAOPTs in Vivo

Triptolide, a natural compound extracted from the traditional Chinese medicine *Tripterygium Wilfordii*, induces cytotoxic tumor cell death and exerts outstanding inhibitory activities against diverse tumors including breast tumor, osteosarcoma, etc. [33, 83]. It has been stated that triptolide can lead to severe toxic and side effects in vivo such as hepatotoxicity, limiting the clinical application of triptolide [83, 84]. In consequence, the safety of HAOPTs containing triptolide should be evaluated in vivo. During the same therapeutic period mentioned above, no loss of body weight was observed in groups except the free triptolide group with apparent weight decrease (Additional file 1: Fig. S11). According to the H&E staining of main organs (Additional file 1: Fig. S12), triptolide generated serious tissue damage and inflammatory cell infiltration in liver while the liver side effects in the DexOPTs group were evidently reduced. HAOPTs, HAOTs and other free drug groups (OA-Met and pro-DHA) exhibited almost no organ injuries, demonstrating the safety of free OA-Met, free pro-DHA and triptolide entrapped in micelles. Furthermore, blood biochemical investigations (AST, ALT and ALP) also exhibited significantly lower liver toxicity of HAOPTs than that of free triptolide (Additional file 1: Fig. S13). In conclusion, the biocompatible HAOPTs improved the clinical application prospect of triptolide.

## Conclusion

In current study, we designed tumor targeting anti-inflammation based micelles (HAOPTs) for EMT inhibition and metastatic tumor therapy. The HDAC inhibitor pro-DHA was synthesized and mixed with the amphiphilic OA-Met to form hybrid micelles by self-assembly, and cytotoxic triptolide was encapsulated in micelles at the same time. HA adsorption on the surface of micelles allowed their obvious cellular uptake in tumor cells, brilliant blood circulation profile and significant targeting effect to orthotopic tumors. Additionally, HAOPTs exhibited excellent cytotoxicity, showed synergistic effects, repressed CSC formation, reduced M2 polarized macrophages, mitigated cell invasive ability, realized AMPK activation and HDAC inhibition, and inverted aberrant expression of E-cadherin, Collagen I and MMP-9 in EMT processes. As a result, HAOPTs successfully suppressed both metastasis formation and primary tumor growth, extending the survival time of tumor bearing mice. The safety application of triptolide and an effective mode for the combination therapy of agents with different anti-inflammatory mechanisms, namely metformin and HDAC inhibitor, could be offered in metastasis treatments. Besides, based on encapsulating agents and polysaccharide decoration, our micelles could be designed as an anti-inflammatory nanoplatform for multifunctional treatments. In brief, we provided proof of concept that the tumor targeting anti-inflammatory nanoplatform would afford a potent, safe and clinical translational option for metastatic tumor therapy via EMT inhibition in combination with tumor growth reduction.

## Supplementary Information

**Additional file 1: Scheme S1** Synthetic route of pro-DHA. **Fig. S1** Characterization of the HDAC inhibitor pro-DHA. The ^1^H-NMR spectrums of **A** propofol, **B** DHA and **C** pro-DHA in CDCl_3_. **D** The ESI-MS result of pro-DHA. **Fig. S2 **TEM images of **A** HAOPTs and **B** OPTs with wider view. **Fig. S3** In vivo fluorescence imaging at 2, 6, 12 and 24 h after intravenous injection with OPTs-DiR, DexOPTs-DiR and HAOPTs-DiR. The white dotted ring represents orthotopic breast tumor tissues. **Fig. S4** Cell cytotoxicity curves of **A** free OA-Met, **B** free pro-DHA, pro-DHA in HAOPs, **C** free triptolide, triptolide in HAOTs, triptolide in DexOPTs and triptolide in HAOPTs on 4T1 cells. Data were represented as mean ± SD (n = 3). **Fig. S5** Quantitative analysis of cancer stem cell-like 4T1 cells with CD44^+^/CD24^-/low^ phenotype in the flow cytometry assay. Data were represented as mean ± SD (n = 3), *P < 0.05, **P < 0.01, ****P < 0.0001. **Fig. S6** Quantitative analysis of M2 macrophage subpopulation in the flow cytometry assay. Data were represented as mean ± SD (n = 3), *P < 0.05, ***P < 0.001, ****P < 0.0001. **Fig. S7** Images of breast tumor tissues stained by immunohistochemical staining with **A** Collagen I and **B** MMP-9. The brown regions represent positive areas of Collagen I or MMP-9, Scale bar, 100 µm. **Fig. S8** The image of ex vivo primary breast tumors. **Fig. S9** Representative images of primary tumor sections analyzed by H&E staining after all treatments. The green dotted line separates necrotic area from normal sites. Scale bar, 200 µm. **Fig. S10** HAOPTs promoted cell apoptosis in primary breast tumor tissues. **A** Representative images of apoptotic tumor tissues (green) after treatments of free OA-Met, free pro-DHA, free triptolide, HAOPs, HAOTs, DexOPTs and HAOPTs. Scale bar, 100 µm. **B** Quantitative analysis of TUNEL positive area. Data were represented as mean ± SD (n = 5), **P < 0.01, ***P < 0.001, ****P < 0.0001. **Fig. S11** Mice body weight curves during the treatment period of free OA-Met, free pro-DHA, free triptolide, HAOPs, HAOTs, DexOPTs and HAOPTs. Data were represented as mean ± SD (n = 5). **Fig. S12** H&E staining images of major organ sections after treatments of free OA-Met, free pro-DHA, free triptolide, HAOTs, DexOPTs and HAOPTs. The black arrows and plus signs in liver section indicated typical liver injuries and representative inflammatory cell infiltration, respectively. Scale bar, 200 µm. **Fig. S13** Blood chemistry analysis of **A** AST, **B** ALT and **C** ALP in mice serum. Data were represented as mean ± SD (n = 5), ****P < 0.0001, compared with the saline group. **Table S1** Characterization of OPTs, DexOPTs and HAOPTs. **Table S2** Loading capacity and encapsulation efficiency of pro-DHA in OPTs, DexOPTs and HAOPTs. **Table S3** Loading capacity and encapsulation efficiency of triptolide in OPTs, DexOPTs and HAOPTs. **Table S4** Optimization of the preparation condition of HAOPTs. **Table S5** Pharmacokinetic parameters of various DiR labeled formulations in SD rats.

## Data Availability

The data that support the conclusions of this study are available from the corresponding author on reasonable request.

## Abbreviations

EMT: Epithelial-mesenchymal transition
HA: Hyaluronic acid
MMP-9: Metalloproteinase-9
CSC: Cancer stem cell
NF-κB: Nuclear factor-κB
AMPK: AMP-activated protein kinase
HDAC: Histone deacetylase
STAT: Signal transducer and activator of transcription
DCC: Dicyclohexylcarbodiimide
DMAP: 4-Dimethylaminopyridine
BHT: Butylated hydroxytoluene
DHA: Docosahexaenoic acid
pro-DHA: Propofol-DHA
DiR: 1,1’-Dioctadecyl-3,3,3’,3’-tetramethyl indotricarbocyanine iodide
MTT: 3-(4,5-Dimethylthiazol-2-yl)-2,5-diphenyltetrazolium bromide
IL-13: Interleukin-13
PE: Phycoerythrin,
FITC: Fluorescein isothiocyanate
p-AMPK: Phospho-AMPK
DMEM: Dulbecco's modified eagle medium
FBS: Fetal bovine serum
bFGF: Basic fibroblast growth factor
EGF: Epidermal growth factor
ESI–MS: Electron spray ionization-mass spectrometry
PDI: Polymer dispersity index
DLS: Dynamic light scattering detector
PBS: Phosphate buffer saline
EE: Encapsulation efficiency
LC: Loading capacity
HPLC: High performance liquid chromatography
CMC: Critical micelle concentration
TEM: Transmission electron microscope
PFA: Paraformaldehyde
CI: Combination index
MSFE: Mammosphere formation efficacy
TLR: Toll-like receptor
H&E: Hematoxylin & eosin
SD: Standard deviation
m/z: Mass-to-charge ratio
Cou: Coumarin-6
AUC_0-24__ h_: Area under curve
t_1/2_: Blood circulation half-life
CL: Clearance
MRT_0-24__ h_: Mean residence time
MPS: Mononuclear phagocyte system
ECM: Extracellular matrix
AST: Aspartate aminotransferase
ALT: Alanine aminotransferase
ALP: Alkaline phosphatase
