# Supplementary material for: Metformin and histone deacetylase inhibitor based anti-inflammatory nanoplatform for epithelial-mesenchymal transition suppression and metastatic tumor treatment
Source: J Nanobiotechnology. 2022 Aug 31;20:394. doi: 10.1186/s12951-022-01592-6 (PMC9429706; doi:10.1186/s12951-022-01592-6)
Supplement: Supplementary file 1 — Additional file 1: Scheme S1 Synthetic route of pro-DHA. Fig. S1 Characterization of the HDAC inhibitor pro-DHA. The 1H-NMR spectrums of A propofol, B DHA and C pro-DHA in CDCl3. D The ESI-MS result of pro-DHA. Fig. S2 TEM images of A HAOPTs and B OPTs with wider view. Fig. S3 In vivo fluorescence imaging at 2, 6, 12 and 24 h after intravenous injection with OPTs-DiR, DexOPTs-DiR and HAOPTs-DiR. The white dotted ring represents orthotopic breast tumor tissues. Fig. S4 Cell cytotoxicity curves of A free OA-Met, B free pro-DHA, pro-DHA in HAOPs, C free triptolide, triptolide in HAOTs, triptolide in DexOPTs and triptolide in HAOPTs on 4T1 cells. Data were represented as mean ± SD (n = 3). Fig. S5 Quantitative analysis of cancer stem cell-like 4T1 cells with CD44+/CD24-/low phenotype in the flow cytometry assay. Data were represented as mean ± SD (n = 3), *P < 0.05, **P < 0.01, ****P < 0.0001. Fig. S6 Quantitative analysis of M2 macrophage subpopulation in the flow cytometry assay. Data were represented as mean ± SD (n = 3), *P < 0.05, ***P < 0.001, ****P < 0.0001. Fig. S7 Images of breast tumor tissues stained by immunohistochemical staining with A Collagen I and B MMP-9. The brown regions represent positive areas of Collagen I or MMP-9, Scale bar, 100 µm. Fig. S8 The image of ex vivo primary breast tumors. Fig. S9 Representative images of primary tumor sections analyzed by H&E staining after all treatments. The green dotted line separates necrotic area from normal sites. Scale bar, 200 µm. Fig. S10 HAOPTs promoted cell apoptosis in primary breast tumor tissues. A Representative images of apoptotic tumor tissues (green) after treatments of free OA-Met, free pro-DHA, free triptolide, HAOPs, HAOTs, DexOPTs and HAOPTs. Scale bar, 100 µm. B Quantitative analysis of TUNEL positive area. Data were represented as mean ± SD (n = 5), **P < 0.01, ***P < 0.001, ****P < 0.0001. Fig. S11 Mice body weight curves during the treatment period of free OA-Met, free pro-DHA, free triptoli [file 12951_2022_1592_MOESM1_ESM.docx]

**Supplementary Data**

**Metformin and Histone Deacetylase Inhibitor Based Anti-inflammatory Nanoplatform for Epithelial-mesenchymal Transition Suppression and Metastatic Tumor Treatment**

Tianze Jiang^1,2^, Laozhi Xie^1^, Songlei Zhou^1^, Yipu Liu^1^, Yukun Huang^1,3^, Ni Mei^4^, Fenfen Ma^1,5^, Jingru Gong^1,5^*, Xiaoling Gao^3^*, Jun Chen^1,6^*

^1^ Shanghai Pudong Hospital & Department of Pharmaceutics, School of Pharmacy, Fudan University, Lane 826, Zhangheng Road, Shanghai 201203, PR China

^2^ Key Laboratory of Marine Drugs, Ministry of Education, Shandong Key Laboratory of Glycoscience and Glycotechnology, School of Medicine and Pharmacy, Ocean University of China, 5 Yushan Road, Qingdao 266003, PR China

^3^ Department of Pharmacology and Chemical Biology, Shanghai Jiao Tong University School of Medicine, 280 South Chongqing Road, Shanghai 200025, PR China

^4^ Shanghai Center for Drug Evaluation and Inspection, Lane 58, HaiQv Road, Shanghai 201210, PR China

^5^ Department of Pharmacy, Shanghai Pudong Hospital, Fudan University Pudong Medical Center, 2800 Gongwei Road, Shanghai 201399, PR China

^6^ Key Laboratory of Smart Drug Delivery, Ministry of Education, School of Pharmacy, Fudan University, Lane 826, Zhangheng Road, Shanghai 201203, PR China

*Corresponding authors:

Jun Chen

Shanghai Pudong Hospital & Department of Pharmaceutics, School of Pharmacy, Fudan University

Key Laboratory of Smart Drug Delivery, Ministry of Education, School of Pharmacy, Fudan University

Lane 826, Zhangheng Road, Shanghai 201203, PR China

E-mail: chenjun@fudan.edu.cn

Xiaoling Gao

Department of Pharmacology and Chemical Biology, Shanghai Jiao Tong University School of Medicine

280 South Chongqing Road, Shanghai 200025, PR China

E-mail: shellygao1@sjtu.edu.cn

Jingru Gong

Shanghai Pudong Hospital & Department of Pharmaceutics, School of Pharmacy, Fudan University, Lane 826, Zhangheng Road, Shanghai 201203, PR China

Department of Pharmacy, Shanghai Pudong Hospital, Fudan University Pudong Medical Center, 2800 Gongwei Road, Shanghai 201399, PR China

E-mail: jingru_gong001@163.com





**Scheme S1** Synthetic route of pro-DHA.


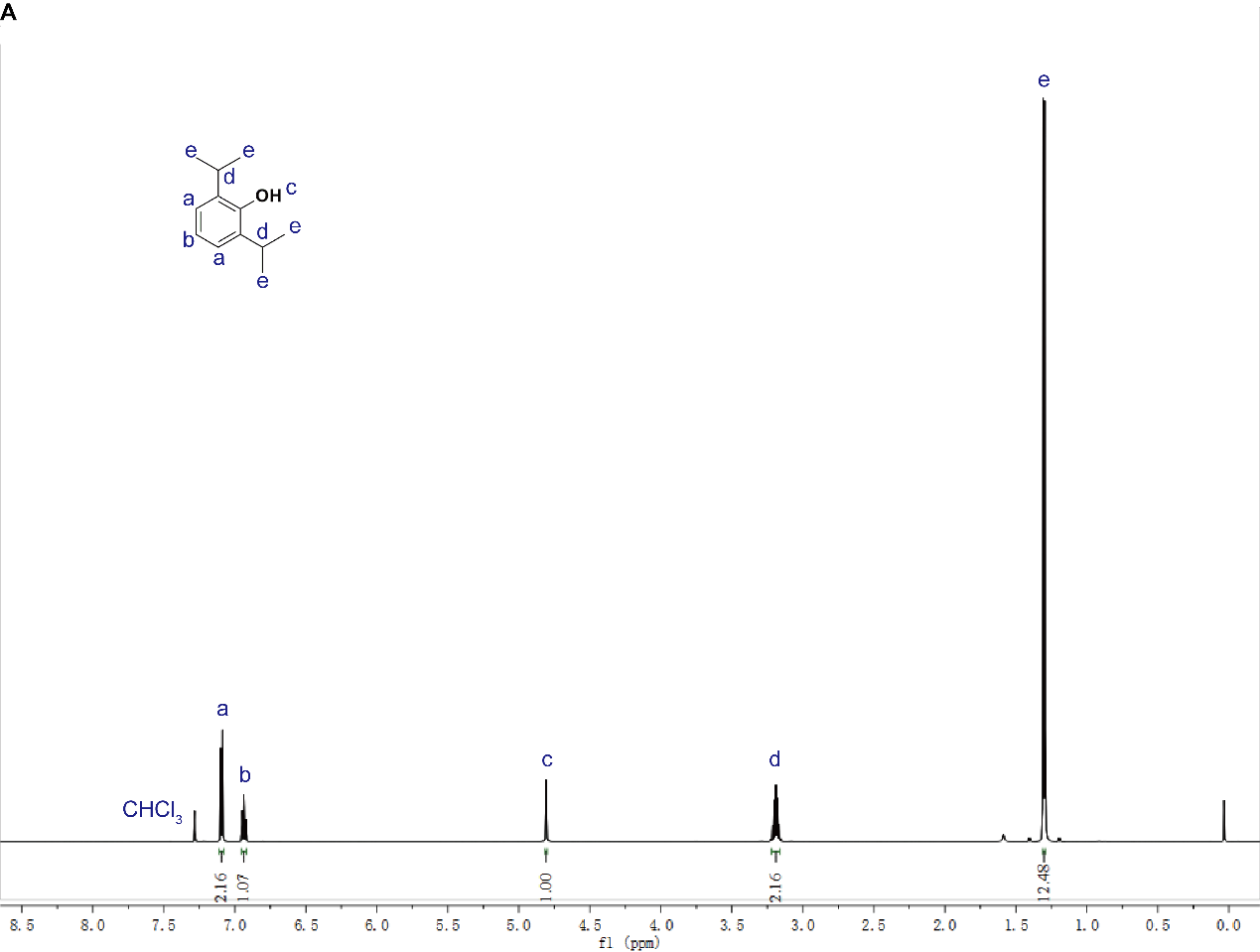


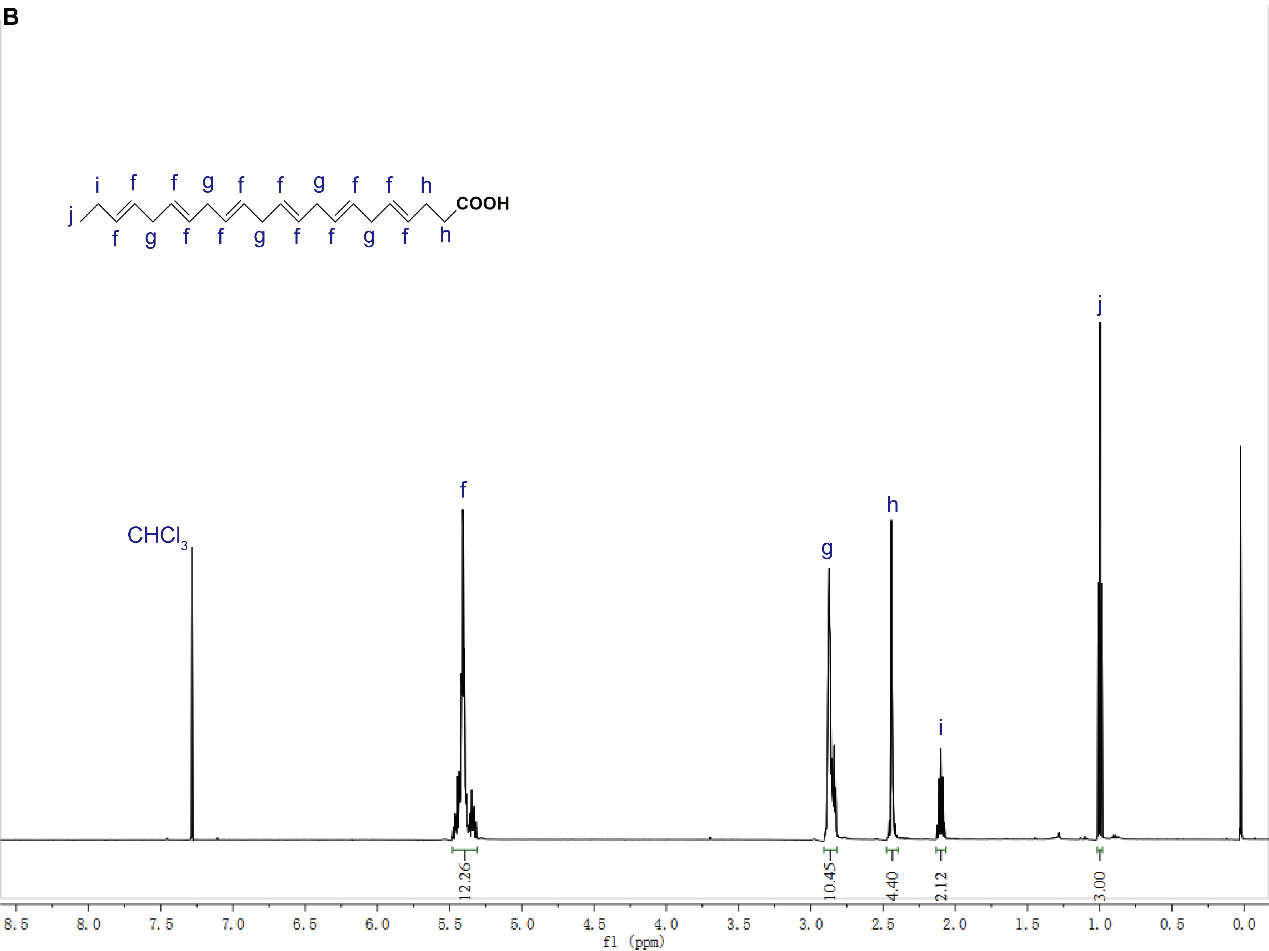


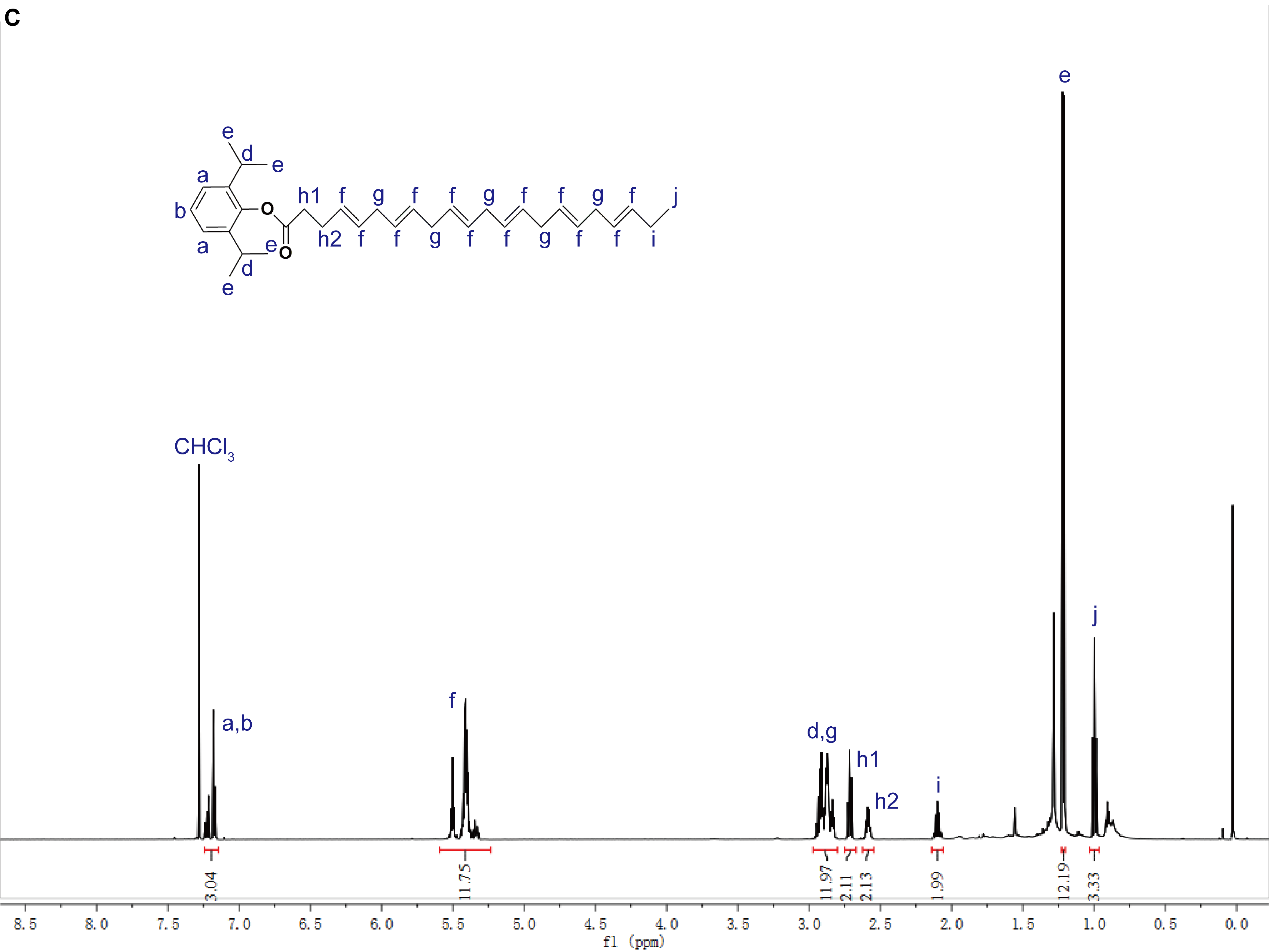


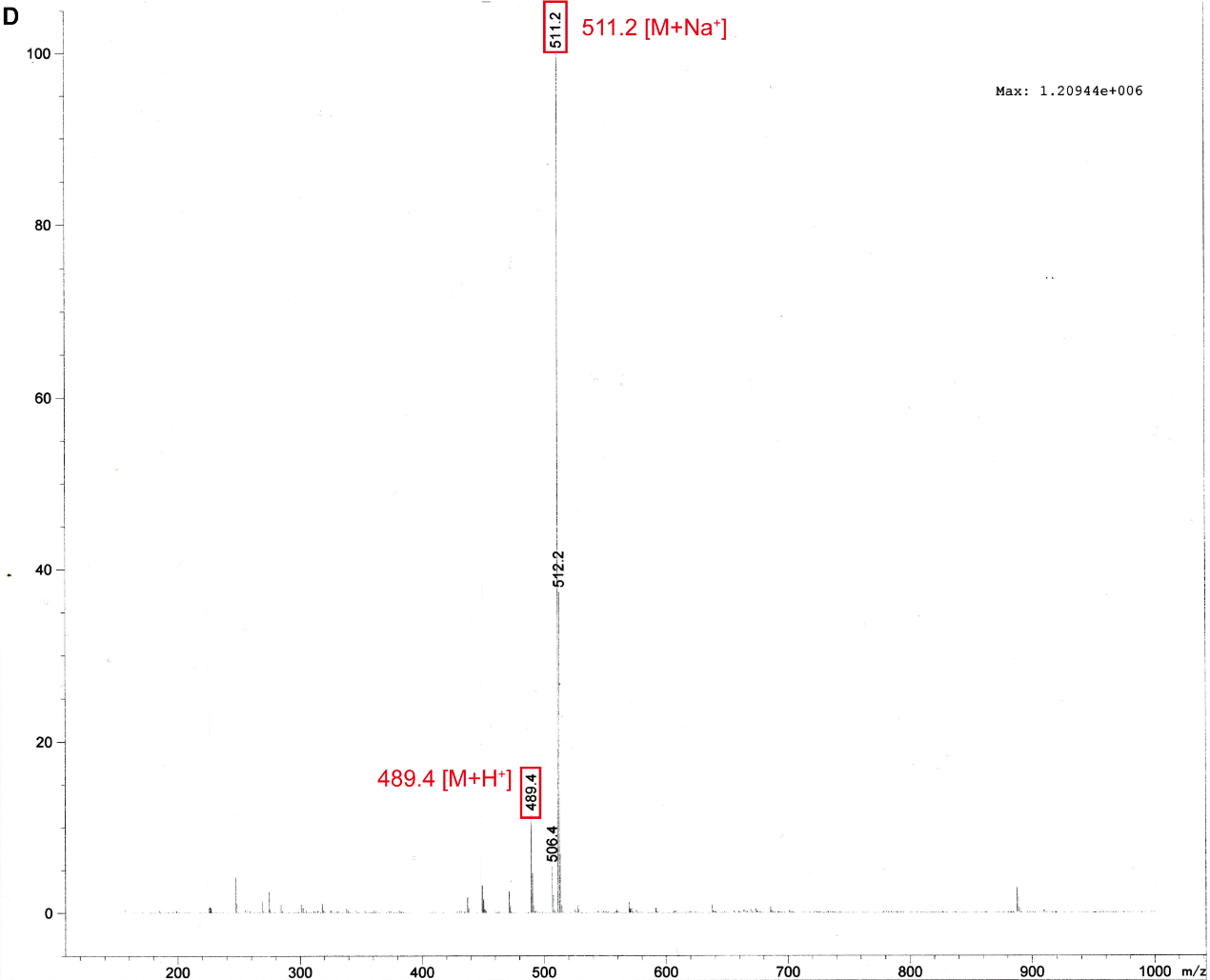


**Fig. S1** Characterization of the HDAC inhibitor pro-DHA. The ^1^H-NMR spectrums of **A** propofol, **B** DHA and **C** pro-DHA in CDCl_3_. **D** The ESI-MS result of pro-DHA.


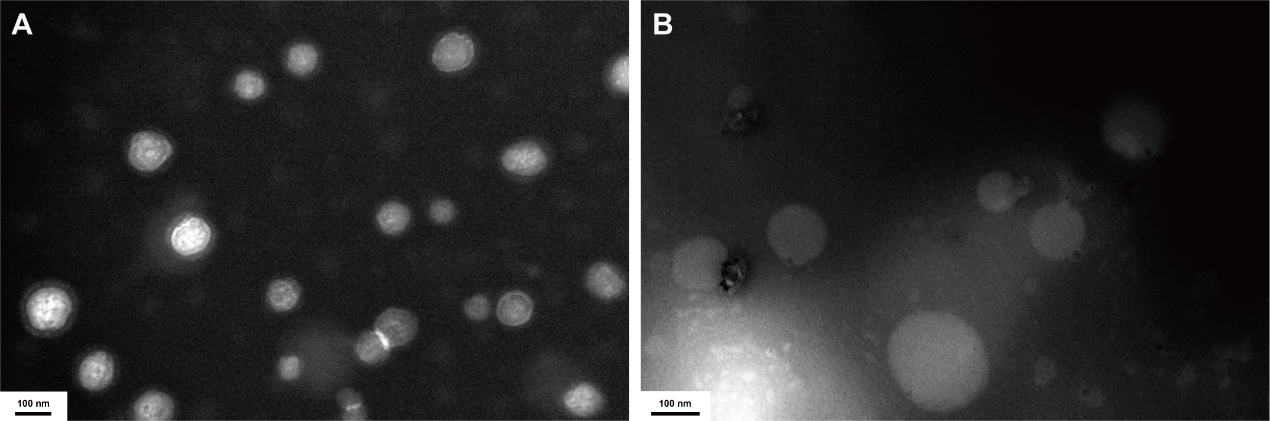


**Fig. S2** TEM images of **A** HAOPTs and **B** OPTs with wider view.


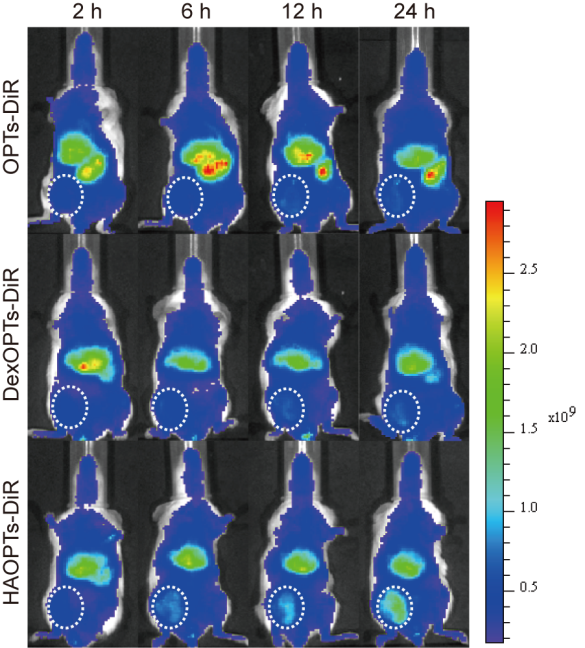


**Fig. S3** In vivo fluorescence imaging at 2, 6, 12 and 24 h after intravenous injection with OPTs-DiR, DexOPTs-DiR and HAOPTs-DiR. The white dotted ring represents orthotopic breast tumor tissues.


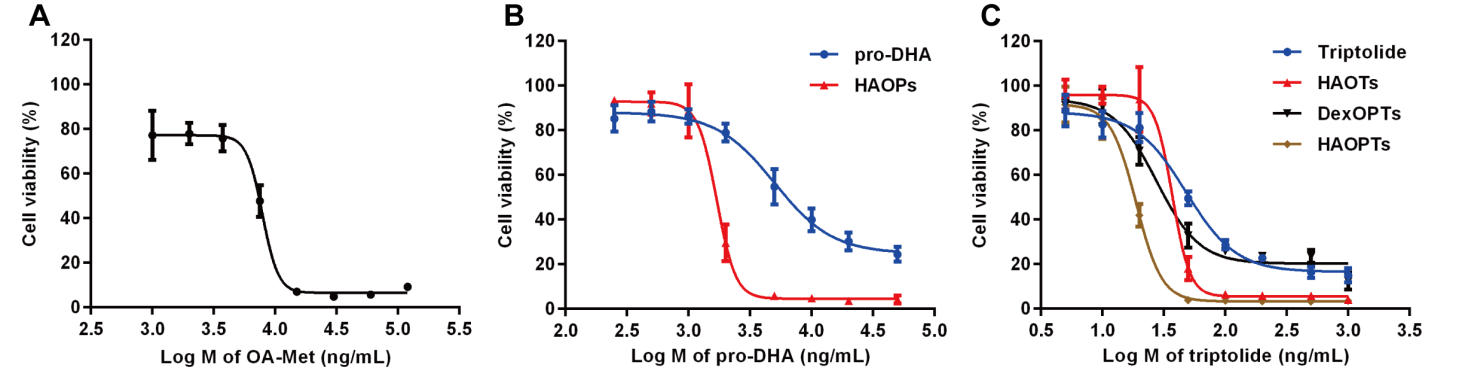


**Fig. S4** Cell cytotoxicity curves of **A** free OA-Met, **B** free pro-DHA, pro-DHA in HAOPs, **C** free triptolide, triptolide in HAOTs, triptolide in DexOPTs and triptolide in HAOPTs on 4T1 cells. Data were represented as mean ± SD (n = 3).





**Fig. S5** Quantitative analysis of cancer stem cell-like 4T1 cells with CD44^+^/CD24^-/low^ phenotype in the flow cytometry assay. Data were represented as mean ± SD (n = 3), **P* < 0.05, ***P* < 0.01, *****P* < 0.0001.





**Fig. S6** Quantitative analysis of M2 macrophage subpopulation in the flow cytometry assay. Data were represented as mean ± SD (n = 3), **P* < 0.05, ****P* < 0.001, *****P* < 0.0001.


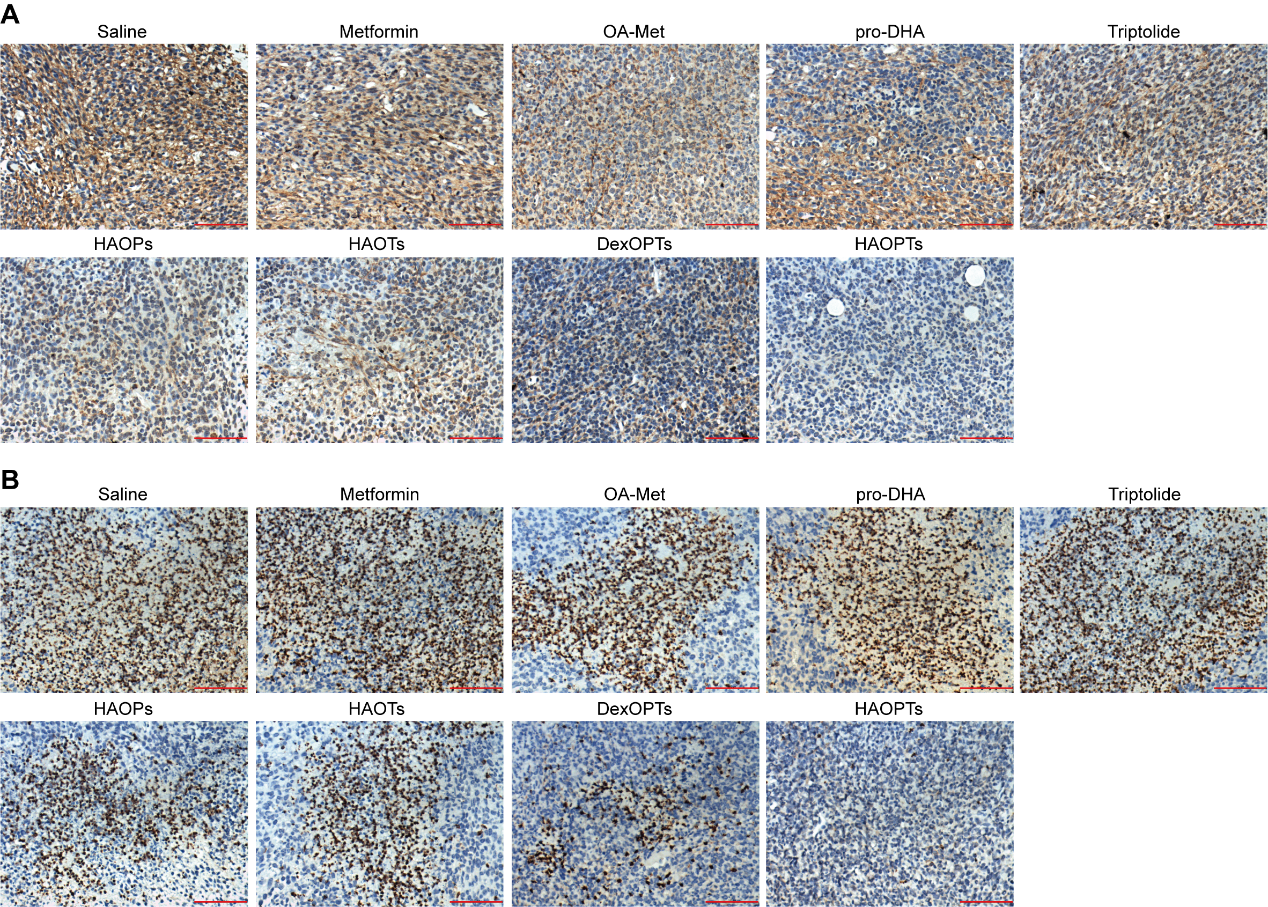


**Fig. S7** Images of breast tumor tissues stained by immunohistochemical staining with **A** Collagen I and **B** MMP-9. The brown regions represent positive areas of Collagen I or MMP-9, Scale bar, 100 µm.


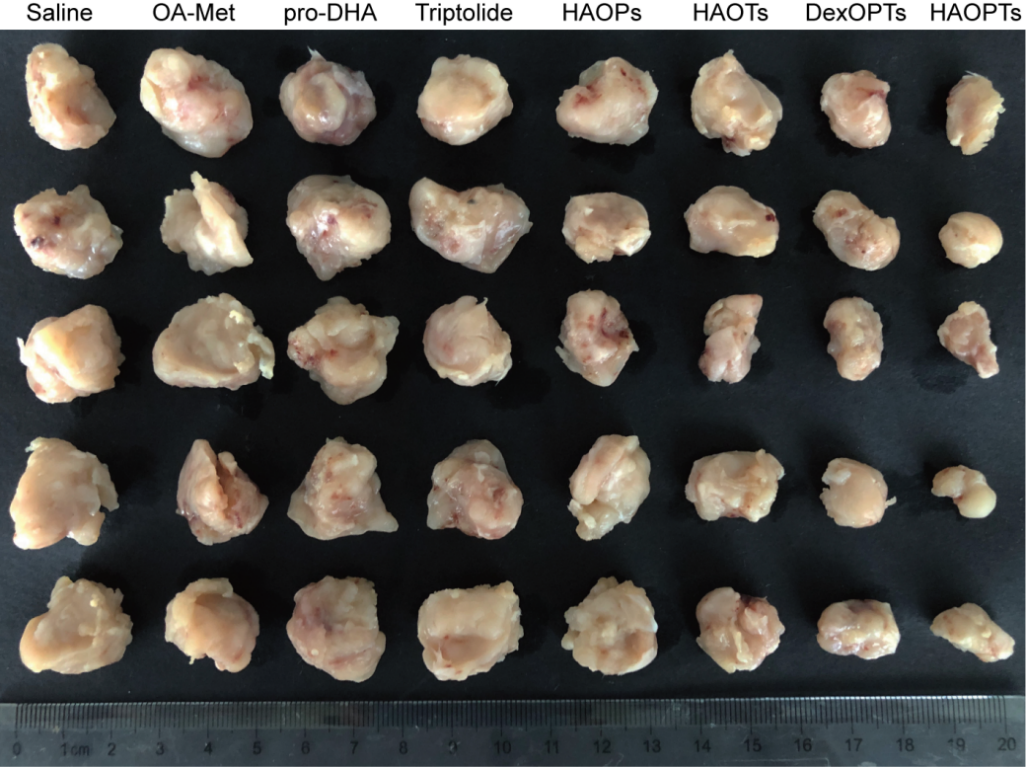


**Fig. S8** The image of ex vivo primary breast tumors.


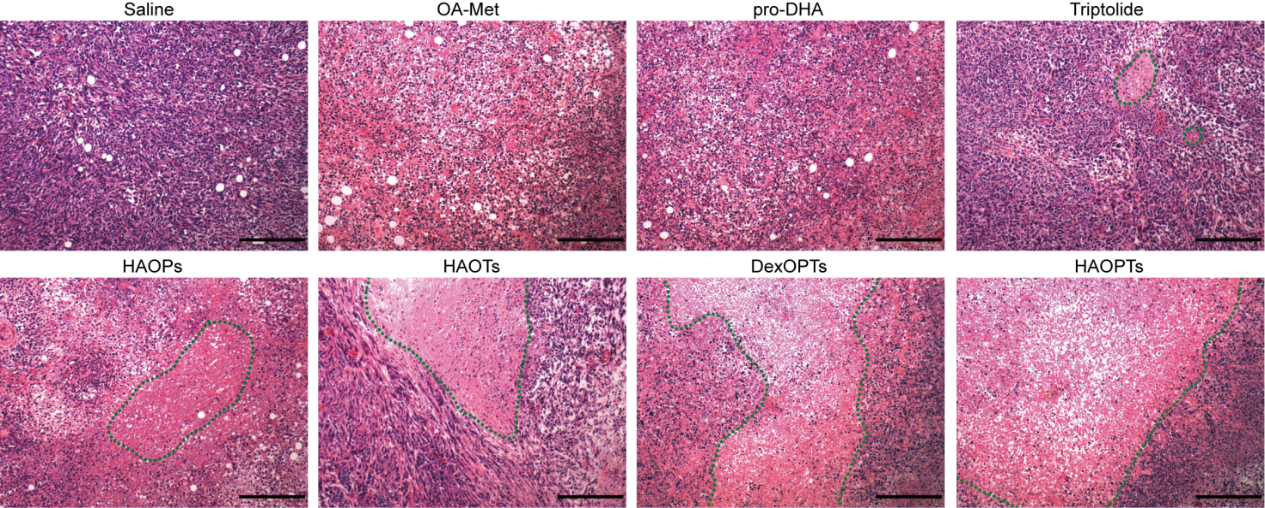


**Fig. S9** Representative images of primary tumor sections analyzed by H&E staining after all treatments. The green dotted line separates necrotic area from normal sites. Scale bar, 200 µm.


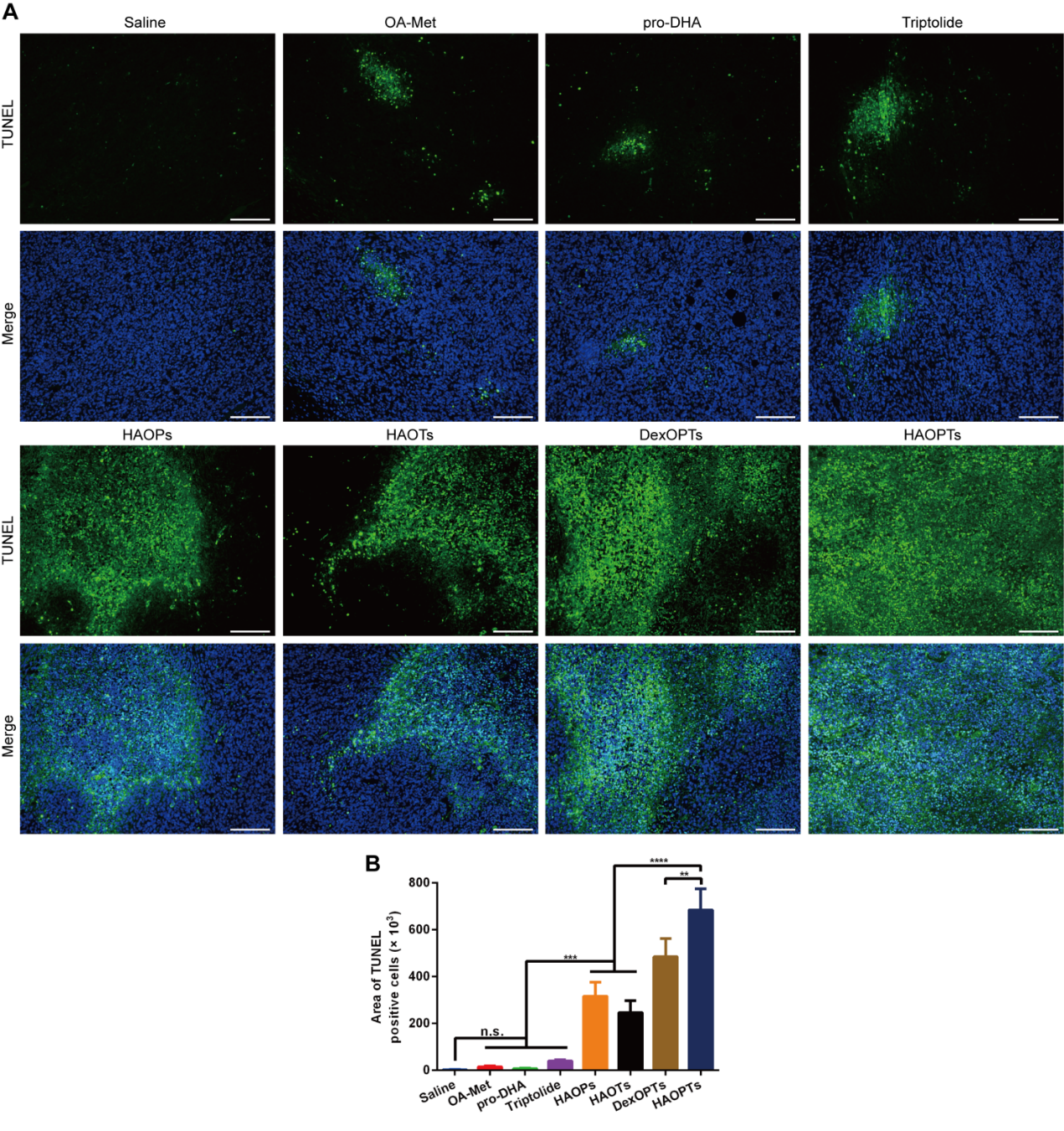


**Fig. S10** HAOPTs promoted cell apoptosis in primary breast tumor tissues. **A** Representative images of apoptotic tumor tissues (green) after treatments of free OA-Met, free pro-DHA, free triptolide, HAOPs, HAOTs, DexOPTs and HAOPTs. Scale bar, 100 µm. **B** Quantitative analysis of TUNEL positive area. Data were represented as mean ± SD (n = 5), ***P* < 0.01, ****P* < 0.001, *****P* < 0.0001.





**Fig. S11** Mice body weight curves during the treatment period of free OA-Met, free pro-DHA, free triptolide, HAOPs, HAOTs, DexOPTs and HAOPTs. Data were represented as mean ± SD (n = 5).


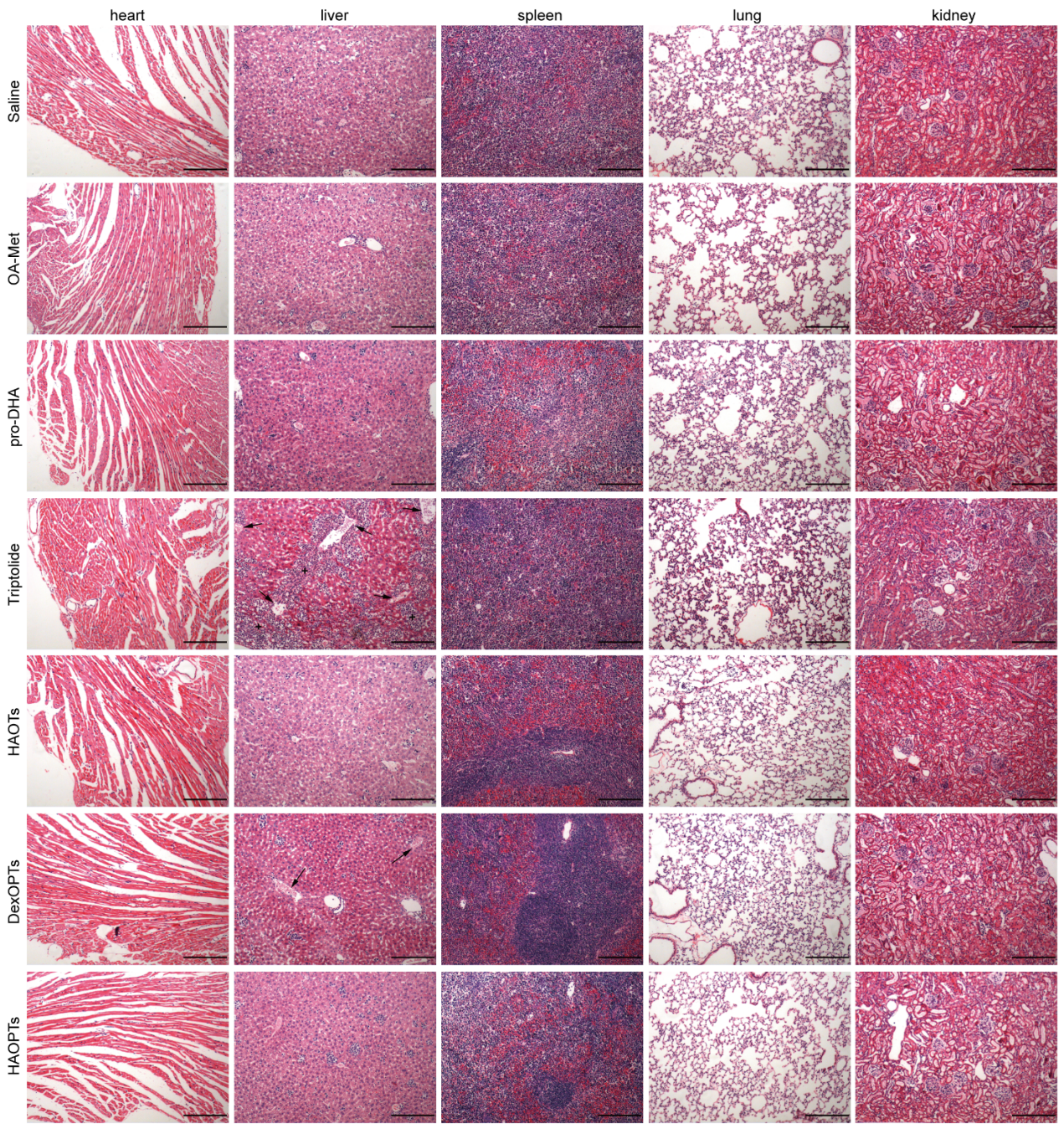


**Fig. S12** H&E staining images of major organ sections after treatments of free OA-Met, free pro-DHA, free triptolide, HAOTs, DexOPTs and HAOPTs. The black arrows and plus signs in liver section indicated typical liver injuries and representative inflammatory cell infiltration, respectively. Scale bar, 200 µm.


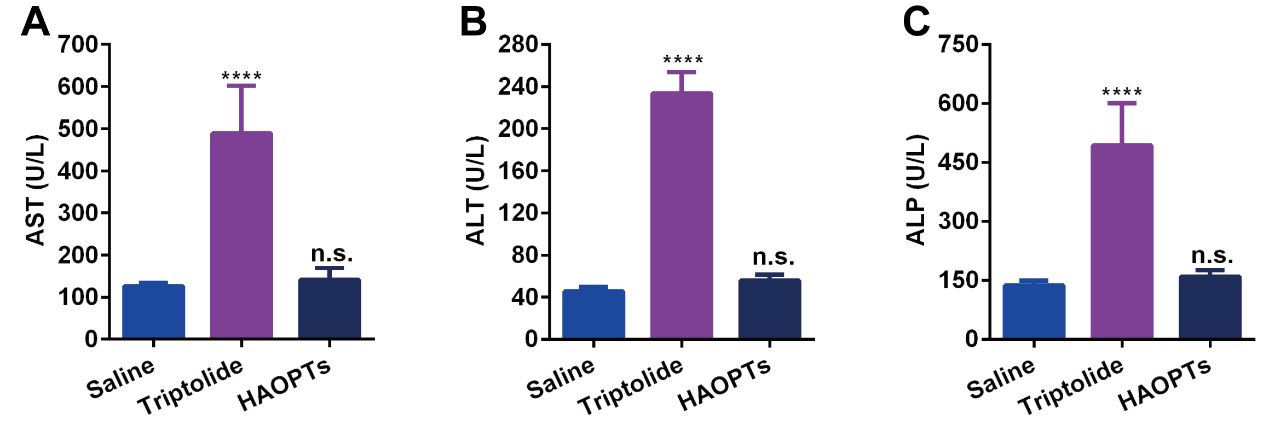


**Fig. S13** Blood chemistry analysis of **A** AST, **B** ALT and **C** ALP in mice serum. Data were represented as mean ± SD (n = 5), *****P* < 0.0001, compared with the saline group.

**Table S1** Characterization of OPTs, DexOPTs and HAOPTs.

| Micelles | Particle size (nm) | PDI | Zeta potential (mV) |
| --- | --- | --- | --- |
| OPTs | 140.7 ± 3.2^a)^ | 0.210 ± 0.014 | 46.4 ± 3.6 |
| DexOPTs | 175.3 ± 2.6 | 0.124 ± 0.019 | -19.1 ± 2.2 |
| HAOPTs | 170.2 ± 2.4 | 0.124 ± 0.016 | -19.6 ± 3.2 |

^a)^ Data were represented as mean ± SD (n = 3).

**Table S2** Loading capacity and encapsulation efficiency of pro-DHA in OPTs, DexOPTs and HAOPTs.

|  | OPTs | DexOPTs | HAOPTs |
| --- | --- | --- | --- |
| Loading capacity (%) | 20.8 ± 0.5^a)^ | 8.8 ± 0.5 | 9.4 ± 0.4 |
| Encapsulation efficiency (%) | 77.0 ± 1.6 | 64.9 ± 2.1 | 69.1 ± 2.9 |

^a)^ Data were represented as mean ± SD (n = 3).

**Table S3** Loading capacity and encapsulation efficiency of triptolide in OPTs, DexOPTs and HAOPTs.

|  | OPTs | DexOPTs | HAOPTs |
| --- | --- | --- | --- |
| Loading capacity (%) | 5.16 ± 0.17^a)^ | 2.32 ± 0.21 | 2.45 ± 0.20 |
| Encapsulation efficiency (%) | 91.1 ± 1.7 | 82.0 ± 1.8 | 86.5 ± 2.0 |

^a)^ Data were represented as mean ± SD (n = 3).

**Table S4** Optimization of the preparation condition of HAOPTs.

| Micelle number | The mass ratio of triptolide to pro-DHA plus OA-Met (%) | Particle size (nm) | PDI |
| --- | --- | --- | --- |
| 1 | 4% (3 : 10)^a)^ | 144.4 ± 1.7^b)^ | 0.282 ± 0.034 |
| 2 | 6% (3 : 10) | 155.9 ± 2.1 | 0.373 ± 0.031 |
| 3 | 8% (3 : 10) | 165.9 ± 3.9 | 0.346 ± 0.033 |
| 4 | 4% (2 : 5) | 142.1 ± 2.3 | 0.241 ± 0.029 |
| **5** | **6% (2 : 5)** | **140.7 ± 3.2** | **0.210 ± 0.014** |
| 6 | 8% (2 : 5) | 171.3 ± 1.2 | 0.318 ± 0.015 |
| 7 | 4% (1 : 2) | precipitation | precipitation |
| 8 | 6% (1 : 2) | precipitation | precipitation |
| 9 | 8% (1 : 2) | precipitation | precipitation |

^a)^ The ratio in parentheses was the mass ratio of pro-DHA to OA-Met. ^b)^ Data were represented as mean ± SD (n = 3).

**Table S5** Pharmacokinetic parameters of various DiR labeled formulations in SD rats.

| Formulations | AUC_0-24h_ (ng/mL*h) | t_1/2_ (h) | CL (mL/h*kg) | MRT_0-24h_ (h) |
| --- | --- | --- | --- | --- |
| Free DiR | 706.32 ± 25.31^a)^ | 3.72 ± 0.09 | 77.51 ± 2.83 | 3.56 ± 0.12 |
| OPTs-DiR | 3888.97 ± 83.56 | 4.09 ± 0.16 | 14.07 ± 0.31 | 5.54 ± 0.11 |
| DexOPTs-DiR | 9907.26 ± 1322.77^**b)^ | 13.12 ± 2.73^**^ | 5.58 ± 0.71^***^ | 7.67 ± 0.09^****^ |
| HAOPTs-DiR | 10488.12 ± 1960.13^***^ | 14.65 ± 4.68^**^ | 5.35 ± 1.12^***^ | 7.81 ± 0.24^****^ |

^a)^ Data were represented as mean ± SD (n = 3); ^b)^ ***P* < 0.01, ****P* < 0.001, *****P* < 0.0001 significantly different with that of OPTs-DiR.
